# The value of information gathering in phage–bacteria warfare

**DOI:** 10.1093/pnasnexus/pgad431

**Published:** 2024-01-09

**Authors:** Yuval Dahan, Ned S Wingreen, Yigal Meir

**Affiliations:** Department of Physics, Ben Gurion University of the Negev, Beer Sheva, 84105, Israel; Department of Molecular Biology, Princeton University, Princeton, NJ 08544, USA; Lewis-Sigler Institute for Integrative Genomics, Princeton University, Princeton, NJ 08544, USA; Department of Physics, Ben Gurion University of the Negev, Beer Sheva, 84105, Israel; Department of Physics, Princeton University, Princeton, NJ 08544, USA

**Keywords:** phage, bacteria, infection strategy, lysis, lysogen

## Abstract

Phages—viruses that infect bacteria—have evolved over billions of years to overcome bacterial defenses. Temperate phage, upon infection, can “choose” between two pathways: lysis—in which the phage create multiple new phage particles, which are then liberated by cell lysis, and lysogeny—where the phage’s genetic material is added to the bacterial DNA and transmitted to the bacterial progeny. It was recently discovered that some phages can read information from the environment related to the density of bacteria or the number of nearby infection attempts. Such information may help phage make the right choice between the two pathways. Here, we develop a theoretical model that allows an infecting phage to change its strategy (i.e. the ratio of lysis to lysogeny) depending on an outside signal, and we find the optimal strategy that maximizes phage proliferation. While phages that exploit extra information naturally win in competition against phages with a fixed strategy, there may be costs to information, e.g. as the necessary extra genes may affect the growth rate of a lysogen or the burst size of new phage for the lysis pathway. Surprisingly, even when phages pay a large price for information, they can still maintain an advantage over phages that lack this information, indicating the high benefit of intelligence gathering in phage–bacteria warfare.

**Significance Statement**

The warfare between bacteria and phage, the most abundant organisms on the planet, has been raging for billions of years. During this time, both sides have developed sophisticated attack and defense strategies. Here, we explore a recently demonstrated phage strategy, in which they garner information about the environment and adapt their infection strategy accordingly. Within a theoretical model, we demonstrate the potentially high value of such information. A deeper understanding of information gathering by phage may help us harness phage for medical purposes.

## Introduction

**Fig. 1.**
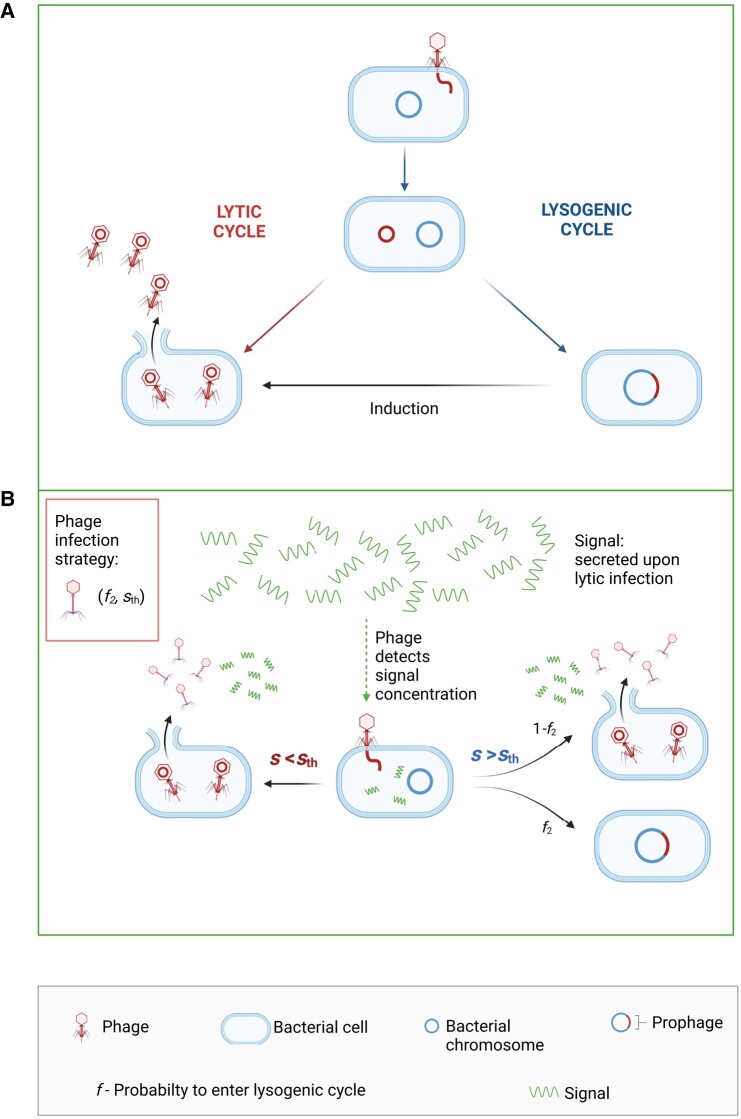
Description of the phage–bacteria system and the model. A) Lytic and lysogenic life cycles of temperate phage. Each phage, after infecting a bacterium, follows either the lytic life cycle (left), where the phage replicates itself and the new phage particles are then released into the medium, or the lysogenic life cycle (right), where its DNA is incorporated into the bacterial DNA as a prophage (5). B) In the model, when a phage can read an input signal from the environment, it can change its strategy depending on whether the outside signal *s* is smaller or larger than a threshold $s_{\mathrm{th}}$, from always lytic ($f_{1}=0$) to lysogenic with probability $f_{2}$ (5).

Warfare between bacteria and phage, the most abundant organisms on the planet, has been raging for billions of years and has played an important role in biodiversity (1–4). The coevolution of bacteria and phage has led to a plethora of defensive and offensive strategies for both phage and bacteria. One common phage strategy is that of temperate phage which, upon infecting bacteria, can choose between two life cycles: a lytic cycle or a lysogenic cycle. In the lytic cycle, the phage takes over the bacterial machinery and produces multiple phage particles which are then released to the environment, resulting in lysis and death of the bacterial host cell. In the lysogenic cycle, the phage’s genetic material is incorporated into the host genome, passing to the cell’s progeny, and may induce production of new phage in the future. Both life cycles are presented in Fig. 1A. Since a lysogenic cell is generally immune to further infections by the same or similar phage, the lysogenic pathway should be advantageous when there is only a small number of uninfected bacteria remaining in the vicinity, whereas the lytic pathway should be preferable when there are still many uninfected bacteria nearby (see Zeng et al. (6) on decision-making at the single-phage level). Thus, phage could gain a significant strategic advantage if they have information on how many uninfected bacteria remain in the environment, or on the ratio of that number to the number of free phage. The importance of such information was discovered early on for *λ* bacteriophage (7), where it was demonstrated that upon simultaneous infection of the cell by multiple phage, the lysogenic pathway was preferred (though recent studies (8, 9) indicate a high lysogeny rate can also occur at high cell density).

Recently, there have been discoveries of phage employing even more sophisticated mechanisms to obtain such crucial intelligence. Erez et al. (10) showed that some phages release a specific peptide (named arbitrium) upon lytic infection. Later on Aframian et al. (11) showed that arbitrium can be secreted even by dormant phage from inside a lysogenic cell. This peptide is then sensed by newly infecting phage, and affects the lysis–lysogeny branching ratio. A high concentration of peptide in the environment means that multiple infections recently occurred nearby; hence, a large concentration of free phage are likely present. In this case, the phage should prefer the lysogenic pathway, because releasing more phage will probably not lead to further infections. Such dependence of the lysogeny-lysis branching ratio on the peptide concentration has been indeed experimentally demonstrated (10).

Silpe and Bassler (12) demonstrated another intelligence-gathering mechanism employed by phage: eavesdropping on bacteria. Many bacteria communicate information to each other by secreting and sensing small molecules in a process called quorum sensing. Some phages developed the ability to listen in on this quorum-sensing communication (by expressing a similar receptor to the bacterial one), thereby providing the phage direct information on the vicinal density of bacteria. Such information can, of course, influence the lysogeny-lysis decision as well.

In order to gather information from the surroundings and alter their behavior accordingly, phage have to carry additional genes and express them. This comes at a certain cost. For example, an additional gene may slow down the growth rate of a lysogenic cell or decrease the burst size in the lytic pathway. The fact that as far as we know not all phage use such intelligence-gathering mechanisms implies that there might be cases in which this cost is too high, causing an evolutionary disadvantage rather than an advantage.

In this paper, we theoretically investigate, what is the “price,” in terms of lysogenic growth rate or burst size, that phage should be willing to pay in order to gain additional environmental information. We concentrate on the mechanism described in Erez et al. (10), as there are quantitative experimental data available in this case. As we show below, under some relevant conditions, the “value” of this additional information can be rather high, i.e. the phage can pay a high price in terms of, e.g. its lysogenic growth rate compared to the nonintelligence-gathering phage, and still have an advantage (13).

## Model and simulations

As a first step, we develop a model of a bacteria–phage system, described by generalized Lotka–Volterra equations. Similar models have been developed in Refs. (14–18), see Cortes et al. (19) for a review. Uninfected bacteria (concentration *B*) and lysogens (concentration *L*) grow, depending on the concentration of nutrient (*N*), with maximal growth rates $\alpha_{B}$ and $\alpha_{L}$, respectively. Phage (concentration *P*) infect cells at a rate *k* per unit concentration, which for the lytic pathway, results in a burst of *b* new phage (infections of already lysogenic cells result in the death of the phage), while lysogenic cells are spontaneously induced, enter the lytic pathway, and produce a burst of *b* new phage at rate *γ*. (We neglect the time delay between lytic infection and the production of new phage, see Materials and methods and SI Appendix 2.E and Fig. S8.) Each lytic infection and each induction release a bolus of the signal molecule (e.g. a peptide) to the media, with ambient signal concentration denoted by *s*. As shown in Aframian et al. (20), a dormant phage can also release signal from a lysogen. For generality, we included this process with a release rate *β* in the last term in Eq. 5; however, taking that effect into account did not change our results, as can be seen in SI Appendix 2.C and Fig. S6, so we set β=0 in the following. In order to model the intelligence-gathering mechanism, we associate an infection strategy with each type of phage. In principle, strategies are defined by three parameters $\left(f_{1},f_{2},s_{\mathrm{th}}\right)$, where $f_{1}$ and $f_{2}$ denote the probabilities that an infection will lead to lysogeny as opposed to lysis, and $s_{\mathrm{th}}$ denotes a threshold signal concentration, above which the lysogenic pathway probability switches from $f_{1}$ to $f_{2}$. (We also considered a smoother response to the signal concentration, which had minimal effect on the results, see SI Appendix 2.B and Fig. S5.) Since we find that below the threshold, i.e. when the number of uninfected bacteria is large enough, the best strategy is to always choose the lytic pathway ( $f_{1}=0$) (see SI Appendix 2.A and Fig. S4), the different strategies are actually characterized by only two parameters ( $f_{2},s_{\mathrm{th}}$). In addition to these information-based adaptive strategies, we also allow “fixed” strategies that cannot change their lysogeny probability. The adaptive strategy is described in Fig. 1B. The index *i* in Eqs. 1–5 stands for different strategies, where for a given strategy $\left(f_{2},s_{\mathrm{th}}\right)$, the lysogenic pathway probability switches from zero to $f_{2}$ when the signal exceeds $s_{\mathrm{th}}$. In order to be as close to biological conditions as possible, we adapted the parameters from Doekes et al. (18); these parameters appear in Table 1.


(1) dBdt⏟Uninfectedbacteria=αBB∑iPiNN+K⏟Growth−kB∑iPi⏟Infection,

(2) dLidt⏟Lysogens=αLLiNN+K⏟Growth+NN+KfikBPi⏟Lysogenicinfection−γLi⏞Induction,

(3) $$\underset{\mathrm{Phage}}{\underbrace{\frac{dP_{i}}{dt}}}=\underset{\mathrm{Lytic}\;\mathrm{infection}}{\underbrace{(1-f_{i})bkBP_{i}}}-\underset{\mathrm{Absorption}\;\mathrm{upon}\;\mathrm{infection}}{\underbrace{kP_{i}(B+\sum_{i}L_{i})}}+\overset{\mathrm{Induction}}{\overbrace{\gamma bL_{i}}},$$

(4) $$\underset{\mathrm{Nutrient}}{\underbrace{\frac{dN}{dt}}}=-\underset{\mathrm{Consumption}\;\mathrm{by}\;\mathrm{cells}}{\underbrace{\frac{N}{N+K}(\alpha_{B}B+\alpha_{L}\sum_{i}L_{i})}},$$

(5) $$\underset{\mathrm{Signal}\;}{\underbrace{\frac{ds}{dt}}}=\underset{\mathrm{Secreted}\;\mathrm{upon}\;\mathrm{lytic}\;\mathrm{infection}}{\underbrace{kB\sum_{i}P_{i}(1-f_{i})}}+\underset{\mathrm{Secreted}\;\mathrm{upon}\;\mathrm{induction}}{\underbrace{(\gamma +\beta )\sum_{i}L_{i}}}.$$

**Table 1.** Simulation parameters (18, 21, 22).

Parameter	Value	Description
$a_{B}$ (h^−1^)	1	Growth rate of uninfected bacteria
$a_{L}$ (h^−1^)	0.7–1	Growth rate of lysogens
*k* (mL [bacterial equivalents]^−1^ h^−1^)	$10^{-10}$	Infection rate
*K* (bacterial equivalents mL^−1^)	$10^{9}$	Michaelis constant
*b*	$10^{-4}\times 10^{3}$	Burst size
*T* (h)	10	Dilution cycle duration
*γ* (h^−1^)	0.001	Rate of induction

**Fig. 2.**
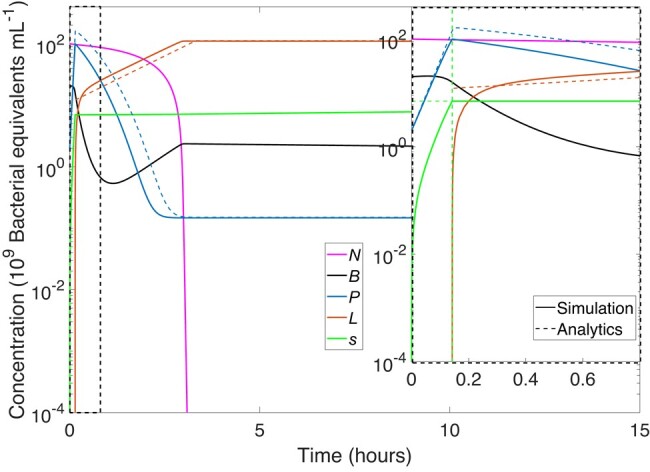
Time evolution of the different populations (main figure) with a zoom (inset) on early times for phage with strategy $\left(f_{2},s_{\mathrm{th}})=(1,7\right)$. At early times, infections of susceptible bacteria (black) only generate new phage particles (blue). When the signal (green) reaches the threshold $s_{\mathrm{th}}=7$ (green horizontal dashed line in inset) at time $t=t_{\mathrm{th}}$ (green vertical dashed line), the lysogenic pathway is chosen with probability $f_{2}=1$, leading to a steep increase in lysogen number (orange). When the nutrients (pink) are exhausted, the system reaches a quasisteady state with only small changes in the populations of phage and lysogens. Solid curves are numerical solutions to Eqs. 1–5, dashed curves are results of an analytical approximation described below (for details see SI Appendix 1.B and 1.C).

Figure 2 depicts the time evolution of the different populations for a specific adaptive strategy $\left(f_{2},s_{\mathrm{th}})=(1,7\right)$. Starting with a mixture of a bacterial population and nutrients with a small number of phage, the number of uninfected bacteria (black) initially grows exponentially, at the maximal growth rate (though this growth is hardly evident in the figure due to its short duration). Since the rate of increase of the number of phage is proportional to the number of bacteria, the phage population (blue) grows double exponentially. Also, since as noted above we take $f_{1}=0$, all successful infection attempts during this time result in lysis. The signal concentration *s* (green) grows due to the increasing number of lytic infections until *s* crosses the threshold (dashed horizontal green line in the inset). For the $f_{2}=1$ strategy shown in Fig. 2, all subsequent successful infections now lead to lysogeny so the number of lysogens (orange) increases. As the number of uninfected bacteria drops considerably, few additional infections occur, and the number of lysogens grows according to the lysogenic growth rate. When the nutrient (pink) is exhausted, the system reaches a quasisteady state, after which changes in the populations are very small, arising only from the slow rate of induction of lysogens.

In order to mimic natural conditions where additional uninfected bacteria or additional nutrients may arrive periodically (23), we perform many cycles of growth according to Eqs. 1–5. At the end of each cycle, the population is diluted by a factor of 100, and new uninfected bacteria and nutrients are added. After many such dilution cycles, the final concentrations of the surviving species stop changing, and our conclusions are based on these ultimate concentrations.

## Results

### Optimal strategies

We expect that over time phage will evolve to adopt the optimal strategy, i.e. the one that leads to the largest number of surviving phage after many generations. Since the time scale for evolution is much longer than the lifetime of a single population of bacteria and phage, we solve Eqs. 1–5 numerically with the aim of identifying the optimal phage strategy. We thus begin by finding the strategy parameters (i.e. $f_{2}$ and $s_{\mathrm{th}}$) that allow a specific adaptive strategy to dominate the phage population after many dilution cycles (such that the population behaves identically during each dilution cycle)—we define this strategy as the optimal strategy. To find this strategy, we insert into the simulation 231 strategies (i.e. all combinations of ( $f_{2},s_{\mathrm{th}})$ in the relevant parameter regime where $f_{2}\in [0,1]$ with step size 0.1 and $s_{\mathrm{th}}\in [0,s_{\max}=20]$ with step size 1). All 231 strategies are inserted together into the simulation, and the one that dominates at long times is considered to be the optimal strategy. We next follow the same procedure to find the optimal fixed strategy (which is equivalent to taking $s_{\mathrm{th}}=0$) (for details of the optimization process see Materials and methods). For the set of parameters given in Table 1, the optimal fixed strategy is f=0.1, while the optimal adaptive strategy is $\left(f_{2},s_{\mathrm{th}})=(1,13\right)$. We note that these findings, i.e. that the optimal adaptive strategy is a transition from fully lytic to full lysogenic infection, and that the optimal fixed strategy has a low lysogen fraction, are in agreement with the results in Doekes et al. (18).

**Fig. 3.**
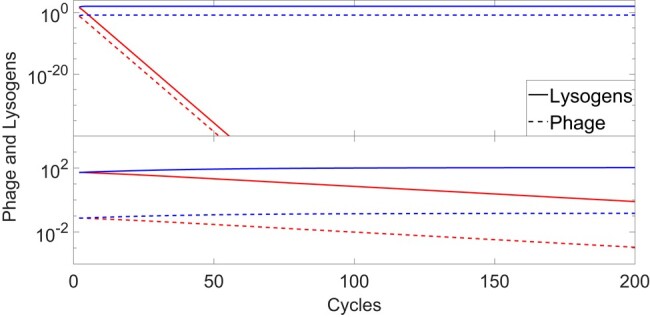
Superiority of the optimal adaptive strategy. Upper panel: The concentration of lysogens (solid curves) and of free phage (dashed curves) at the end of each cycle, as a function of the number of cycles for a competition between the best adaptive strategy, $\left(f_{2},s_{\mathrm{th}})=(1,13\right)$ in blue, and the best-fixed strategy f=0.1 in red. Lower panel: Same for the best adaptive strategy and an inferior adaptive strategy $\left(f_{2},s_{\mathrm{th}})=(1,12\right)$. In both cases, the nonoptimal strategy eventually decays, while the optimal strategy survives. All concentrations are in units of ( $10^{9}$ bacterial equivalents mL ^−1^).

As a first step to quantify the value of information to phage, we compete these two optimal strategies against each other. Since fixed strategies are a subset of adaptive strategies, clearly the optimal fixed strategy cannot outcompete the optimal adaptive strategy. Indeed, Fig. 3A demonstrates that the population of the fixed-strategy phage decays exponentially with the number of cycles, while that of the optimal adaptive strategy stays high. Similarly, Fig. 3B shows a competition between the optimal adaptive strategy and another adaptive strategy $\left(f_{2},s_{\mathrm{th}})=(1,12\right)$. Similar to Fig. 3A, the population of the inferior adaptive strategy eventually decays, though in this case more slowly.

### Value of information

As mentioned above, an adaptive strategy must collect information from the environment, which requires additional resources, presumably at the expense of other biological processes. In the following, we explore specific examples of what happens if the information gathering comes at a cost either to the growth rate of the lysogens or to the burst size in the lytic pathway. Accordingly, in order to obtain the value of information, we compete the optimal fixed strategy against the optimal adaptive strategy, but with a smaller growth rate or burst size for the latter (to enable this, we must first find the optimal adaptive strategy for each such value).

**Fig. 4.**
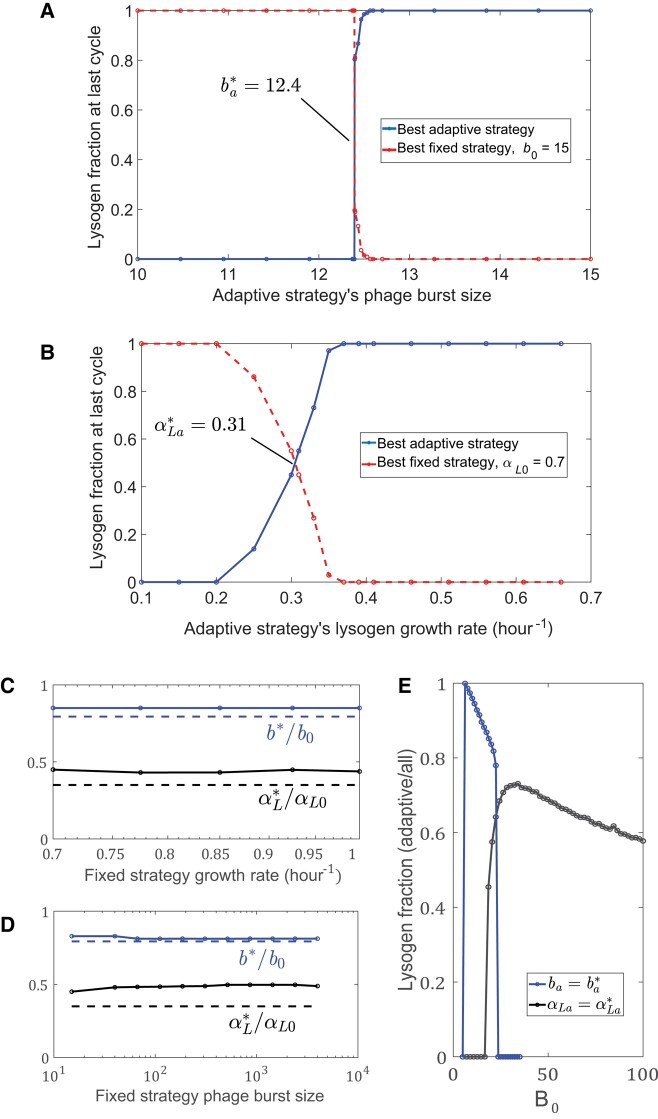
The value of information: Results for competition between the optimal fixed strategy with burst size $b_{0}=15$ and growth rate $\alpha_{L0}=0.7$ and an optimal adaptive strategy whose burst size $b_{a}$ A) or lysogen growth rate $\alpha_{\mathrm{La}}$ B) is varied. (For each burst size and growth rate value, the new optimal adaptive strategy was found.) A) When $b_{a}$ becomes smaller than $b_{a}^{*}\approx 12.4$ (with $\alpha_{\mathrm{La}}=\alpha_{L0}$) or B) $\alpha_{\mathrm{La}}$ becomes smaller than $\alpha_{\mathrm{La}}^{*}\approx 0.31$ (with $b_{a}=b_{0}$) the optimal adaptive strategy loses its advantage. C, D) Robustness of the relative crossing points $\alpha_{\mathrm{La}}^{*}/\alpha_{L0}$ and $b_{a}^{*}/b_{0}$ to changes in the values of the fixed strategy burst size $b_{0}$ C) and growth rate $\alpha_{L0}$ D). The points are the numerical results, while the dashed curves indicate the analytic approximation, Eqs. 6 and 7. E) Regions of coexistence between the adaptive and fixed strategies as the concentration of initial bacteria is varied, for the crossing point in A), where the fixed and adaptive strategy burst sizes are $b_{0}$ and $b_{a}^{*}$, respectively, and the crossing point in B), with fixed and adaptive strategy growth rates $\alpha_{L0}$ and $\alpha_{\mathrm{La}}^{*}$. While there is coexistence in both cases, the region of coexistence is much narrower for different burst sizes than for different growth rates.

Figure 4A shows the results of competitions between the optimal fixed strategy for burst size $b_{0}=15$ and the optimal adaptive strategy over a range of burst sizes $b_{a}$ (with the same lysogenic growth rate). Specifically, we show the end-of-cycle concentration of lysogens of each strategy after the system reaches a steady-state behavior. Clearly when $b_{a}=b_{0}$ the adaptive strategy has an advantage, and indeed the population of lysogens with the fixed strategy (red) eventually vanishes with repeated cycles of growth and dilution. However, as the burst size of the adaptive strategy is decreased, the adaptive strategy starts to lose its advantage over the fixed strategy, until, at a burst size between 12 and 13, it is the adaptive strategy that ultimately loses the competition and eventually vanishes. As the interpolated crossing point is around $b_{a}^{*}=12.4$, the normalized difference $1-b_{a}^{*}/b_{0}≃0.17$ is a measure of the “value of information,” i.e. by what fraction the adaptive strategy can reduce its burst size and still retain an advantage over the fixed strategy. As the growth rate of the number of phage is proportional to the burst size, we find, surprisingly, that in this model the phage should be willing to pay a cost of up to almost 20% reduced growth rate in order to obtain information about the changing environment. Similarly, Fig. 4B depicts the value of information in terms of the reduced lysogenic growth rate of the adaptive strategy. Even more surprisingly, in this case, the value of information is $1-\alpha_{\mathrm{La}}^{*}/\alpha_{L0}≃0.55$, with $\alpha_{L0}$ the lysogenic growth rate of the fixed strategy, i.e. the adaptive strategy can lower its lysogen’s growth rate by more than a factor of 2, and still have an advantage over the fixed strategy, indicating the high value of the collected information.

One obvious question is how sensitive these values are to the other parameter choices. Figure 4C and D shows the values of information $b_{a}^{*}/b_{0}$ and $\alpha_{\mathrm{La}}^{*}/\alpha_{L0}$ as functions of different burst sizes ($b_{0}$) and growth rates ( $\alpha_{L0}$) of the fixed strategy. The value of information is quite robust to changes of these parameters.

## Analytical approximation

To gain more insight into these results, we derive an approximate analytical solution to Eqs. 1–5, based on the observation that immediately following dilution, the rate of growth of the phage population is extremely fast compared to the bacterial growth rate, but the phage population remains small enough not to substantially reduce the population of susceptible bacteria. Accordingly, one can assume a constant bacterial population during this initial phase of rapid phage growth (see SI Appendix 1.B and 1.C for full details). In Fig. 2, we compare the results for the phage and lysogen concentrations based on this approximation to the full numerical solution. We see that the approximation captures quantitatively the time evolution and the concentrations of phage and lysogens over the course of a dilution cycle. Moreover, from the analytic solution, it is possible to determine the crossing point between dominance by an adaptive strategy and dominance by a fixed strategy with a larger burst size or lysogen growth rate. The procedure involves the following steps: (i) Given an arbitrary fixed strategy (with growth rate $\alpha_{L0}$ and burst size $b_{0}$), we check whether this fixed strategy can be invaded by any other fixed strategy, which allows us to find the optimal, noninvasible fixed strategy $f_{0}$. For the biologically relevant parameters used in the simulations, this gives $f_{\mathrm{opt}}≃0.08$, in good agreement with the simulations. (ii) We next consider the invasibility of this optimal fixed strategy by an adaptive strategy with a burst size $b_{a}$ (and the same $\alpha_{L0}$), or by an adaptive strategy with a lysogenic growth rate $\alpha_{\mathrm{La}}$ (and the same $b_{0}$). By maximizing the number of the resulting lysogens of the adaptive strategy at the end of the dilution cycle, we find, for each $b_{a}$ and for each $\alpha_{\mathrm{La}}$, its optimal adaptive parameters $\left(f_{2},s_{\mathrm{th}}\right)$. (iii) Lastly, we check the invasibility of the optimal fixed strategy by the optimized adaptive strategy as a function of $b_{a}$ or $\alpha_{\mathrm{La}}$ of the latter. This allows us to determine the crossing-point values $b_{a}^{*}$ and $\alpha_{\mathrm{La}}^{*}$ below which the adaptive strategy is no longer able to invade the fixed strategy (see SI Appendix 1.C for the full derivation):


(6) $$\frac{b_{a}^{*}}{b_{0}}=(1-f_{0})\frac{\log\;(ef_{0}b_{a}^{*}B_{0}/P_{0})}{\log\;(B_{0}b_{0}(1-f_{0})/P_{0})},$$

(7) $$\frac{\alpha_{\mathrm{La}}^{*}}{\alpha_{L0}}=1+\frac{\left(1-f_{0})\log\;(ef_{0}b_{0}B_{0}/P_{0})-\log\;(B_{0}b_{0}(1-f_{0})/P_{0}\right)}{\left(1-f_{0})\log\;(N_{0}/f_{0}B_{0}\right)}.$$

For the parameters used in the simulations and $f_{\mathrm{opt}}≃0.08$, Eqs. 6 and 7 yield the following estimates for the “value of information” $1-b_{a}^{*}/b_{0}≃0.21$ and $1-\alpha_{\mathrm{La}}^{*}/\alpha_{L0}≃0.65$, just slightly higher than the values obtained in the numerical calculations. The analytical results also explain the robustness of these values to the various parameters, as the critical values depend on these parameters only logarithmically, and some of these dependencies cancel between the numerator and the denominator.

## Coexistence of fixed and adaptive strategies

One of the surprising observations, seen in Fig. 4B, is the large crossover region as a function of the adaptive lysogen growth rate $\alpha_{\mathrm{La}}$ between the regime where the fixed strategy dominates and the regime where the adaptive strategy dominates. This is to be compared with Fig. 4A which depicts a very narrow crossover regime with respect to changing the burst size $b_{a}$ of the adaptive strategy. To confirm these observations, we extended our analytical calculations to calculate the stability of the best adaptive strategy with varied $b_{a}$ or $\alpha_{\mathrm{La}}$ to invasion by a fixed strategy, with $b_{0}$ and $\alpha_{L0}$. Indeed, consistent with the numerical calculations, we find that there is a wide region in $\alpha_{\mathrm{La}}$ but only a narrow region in $b_{a}$ where the best-fixed strategy can be invaded by an adaptive strategy and at the same time the best adaptive strategy can be invaded by a fixed strategy.

These observations do not directly mean that two specific strategies can coexist for a finite range of environmental parameters, as each value of $b_{a}$ or $\alpha_{\mathrm{La}}$ denotes a different “species.” Thus, to check for true coexistence, we competed two strategies, one fixed and one adaptive, chosen from the crossover regime, over a range of different environmental conditions such as initial concentrations of nutrients or bacteria. Indeed, as seen in Fig. 4E, we found that there is a wide range of initial bacterial concentrations $B_{0}$ where a fixed strategy with lysogenic growth rate $\alpha_{L0}$ coexists with an adaptive strategy with growth rate $\alpha_{\mathrm{La}}=\alpha_{\mathrm{La}}^{*}$, but only a narrow range of $B_{0}$ values where a fixed strategy with burst size $b_{0}$ coexists with an adaptive strategy with burst size $b_{\mathrm{La}}=b_{\mathrm{La}}^{*}$ (a similar behavior is observed as a function of the initial nutrient concentration, see SI Appendix Fig. S3). One can understand this difference in the context of general ecological theory which dictates that, at a steady state, the number of coexisting strategies cannot exceed the number of resources (24–27). During the initial growth phase, both types of phage “consume” the susceptible bacteria, but the adaptive strategy may expand faster. However, after there are no more uninfected bacteria, the fixed-strategy lysogens grow faster on the remaining nutrients. Thus, the susceptible bacteria and the nutrients serve as two distinct “resources,” which in principle allows for the coexistence of two species. On the other hand, when only the burst sizes of the two strategies are different, then once the susceptible bacteria are consumed, both species grow at the same rate. Thus, in this case, there is only a single resource—the bacteria. The fact that we do see some coexistence in this regime is due to the fact that the bacterial concentration is changing with time, and the system is not in a steady state. The adapting phage is more efficient at converting bacteria to phage at earlier times, while the fixed strategy is more efficient at later times, allowing for coexistence. This argument also explains the qualitative difference between the sharp dependence of the competitiveness of the adapting strategy on burst size (Fig. 4C) vs. the more graded dependence on growth rate (Fig. 4D).

## Summary and discussion

As the war between bacteria and phage has raged, each side has evolved weapons and strategies to give it an advantage over the other. As intelligence gathering is an integral part of warfare, it is not surprising that some phages have invested in obtaining information to guide their attack strategies. Some of these information-gathering methods, such as measuring multiplicity of infection (MOI), are well known from early studies of temperate phage, but recently other information-gathering methods have been uncovered.

Here, we have employed a theoretical model that on one hand is simple enough to analyze, while on the other hand can describe quantitatively phage–bacteria interactions. The main result of this study is that the value of information—the reduction in growth rate or burst size an information-gathering phage can tolerate and still have an advantage—is rather high. The information-gathering phage lysogen, for example, can tolerate a reduction in its growth rate by more than 50%, e.g. due to carrying and expressing extra genes, and still outcompete phages that do not have this information. Rather surprisingly, we find that the value of information is relatively insensitive to changes in the other parameters of the model, and we developed an analytic approach that explains this observation.

The lysogenic growth rate and the burst size (which determines the growth rate of the phage population) underpin exponential processes, and thus small changes to these values may dramatically affect the population size in the long run. Indeed, bacterial populations are large enough that relative growth rate advantages as small as $10^{-5}$ can sweep to fixation (28). Nevertheless, in the present case, the adaptive strategy which gathers and exploits information still maintains an advantage over a fixed strategy even when its growth rate as a lysogen is cut in half. This can be traced back to the significant advantage of the adaptive strategy: when a small population of the best adaptive strategy invades the optimal fixed strategy with the same growth rate, the adaptive population grows by over an order of magnitude more than does the fixed-strategy population for the biologically motivated parameters employed here. Why is the information-gathering strategy so advantageous? During the lytic stage, the concentration of signal molecules is proportional to the concentration of free phage. Thus, employing a signal threshold allows the phage to switch to the lysogenic pathway when the phage concentration crosses a given value, independently of other dynamical parameters such as the bacteria or nutrient concentrations. Moreover, since the lifetime of the population of uninfected bacteria is determined by the phage concentration, this also allows the phage to effectively switch pathways at a specific point during the lifetime of the susceptible bacterial population, allowing the phage to maximize the resulting number of lysogens. This effect is likely to be enhanced in natural conditions, where the amount of new nutrients or new susceptible bacteria may fluctuate. Thus, information gathering may be even more advantageous to phage than our estimates.

While this study indicates that it can be highly worthwhile for phage to gather information, it is only recently that such strategies have been discovered beyond the well-studied MOI. One question is whether there is a hidden cost to such strategies, one that has not been taken into account in our model, or perhaps that recent discoveries of information-gathering phages are just the tip of the iceberg, and, in fact, there may prove to be many more phages that actively gather information. (We exclude the effect of MOI in this study in order to investigate the information-gathering mechanism alone—exploring the two mechanisms together is an interesting topic for future work.) It should be noted that in order for adapting phage to be able to invade and then switch to a different behavior, their initial concentration should be high enough so that at some point the amount of produced signal can cross the threshold. While, as elucidated in the analytical calculation, the important factor is the switching time during the cycle, which depends only logarithmically on the initial phage concentration, this may set a lower limit on the concentration of adapting phage that can invade a nonadapting population.

Optimal switching from lytic to lysogenic infection according to an external signal has recently been explored in several publications, e.g. (18, 29, 30). Here, we extended the investigation to other aspects of the decision mechanism: (i) a measure of how advantageous is the adaptive strategy (i.e. the value of information), (ii) the surprising constancy of that value with respect to model parameters, and (iii) the coexistence of adaptive and nonadaptive strategies. These findings, as far as we know, highlight aspects of decision-making by phage that call for further exploration in theoretical and experimental works.

## Materials and methods

Numerical solutions for Eqs. 1–5 were obtained using MATLAB ode45 function. The parameters used in the simulation are given in Table 1. In the search for optimal strategies, all combinations of $\left(f_{2},s_{\mathrm{th}}\right)$ from the relevant ranges were tested. The relevant ranges were: $f_{2}\in [0,1]$ with step size of 0.1 and $s_{\mathrm{th}}\in [0,20]$ with step size 1. The initial values for each system component are given in Table 2. At the end of each dilution cycle, the population was diluted 100-fold, and new bacteria and nutrients were added, in the same amount as in the initial conditions. While the release of new phage upon lytic infection is not instantaneous, here we neglect this time delay. Taking the delay into account, the result was not affected significantly. For more information, see SI Appendix 2.E and Fig. S8.

**Table 2.** Simulation initial conditions.

Component (bacterial equivalents mL^−1^)	Initial concentration
Uninfected bacteria ($B_{0}$)	$20\times 10^{9}$
Lysogens	0
Phage	$0.1\times B_{0}$
Nutrient	$100\times 10^{9}$
Signal	0

## Supplementary Material

pgad431_Supplementary_Data

## Data Availability

The data that support the findings of this study are available from the corresponding authors upon reasonable request.
