# Supplementary material for: The value of information gathering in phage–bacteria warfare
Source: PNAS Nexus. 2024 Jan 9;3(1):pgad431. doi: 10.1093/pnasnexus/pgad431 (PMC10776245; doi:10.1093/pnasnexus/pgad431)
Supplement: pgad431_Supplementary_Data [file pgad431_supplementary_data.zip › PNASNEXUS-PNASNEXUS-2023-00941R-s01.pdf]

Supplementary Information for

The value of information gathering in phage-bacteria warfare

Yuval Dahan, Ned S. Wingreen, and Yigal Meir

To whom correspondence may be addressed. Email: wingreen@princeton.edu or ymeir@bgu.ac.il

This PDF file includes:

Supplementary text
Figs. S1 to S8
SI References

Supporting Information Text

1. Details of the approximate analytical calculation

In the following, we describe an approximate analytic approach to the solution of Eqs. (1-5) of the main text. This solution sheds light on why the critical values α_L^* and b^* are rather robust, in spite of the exponential dependence of the different populations on the model parameters. As we show below, these critical values depend on these parameters only logarithmically, and in our final expression some of these dependencies cancel between the numerator and the denominator.

Below we describe a standard invasion-stability analysis. We first start with a fixed strategy, optimize it and then check its stability against an invading adapting strategy, which is also optimized. By checking whether the optimal fixed strategy can be invaded successfully by an adapting strategy with a smaller lysogen growth rate or a smaller burst size, we can evaluate the value of information. We then check the stability of an optimal adapting strategy against invasion by a fixed strategy, to check for possible coexistence.

A. A Single Fixed strategy.

Bacteria and phage. We start with a single fixed strategy f_0. Assuming that because of the large burst size the phage population grows quickly on the timescale of bacterial growth, we can initially neglect bacterial growth. Further neglecting the initially small number of lysogens, we have

$$\frac{dB}{dt} = -kB(t)P(t)$$
$$\frac{dP}{dt} = k(1-f_0)bP(t)B(t). \quad [1]$$

Using the fact that $(1-f_0)bB(t) + P(t)$ is independent of time during this period, we find

$$B(t) = \frac{B_0(B_0b(1-f_0)+P_0)}{B_0b(1-f_0)+P_0e^{k(B_0(1-f_0)b+P_0)t}} \approx \frac{B_0^2b(1-f_0)}{B_0b(1-f_0)+P_0e^{kB_0b(1-f_0)t}}$$
$$P(t) = \frac{(B_0b(1-f_0)+P_0)P_0e^{k(B_0(1-f_0)b+P_0)t}}{B_0b(1-f_0)+P_0e^{k(B_0(1-f_0)b+P_0)t}} \approx \frac{B_0b(1-f_0)P_0e^{k(B_0(1-f_0))t}}{B_0b(1-f_0)+P_0e^{kB_0b(1-f_0)t}}, \quad [2]$$

where the approximation holds for the biologically relevant case $\frac{P_0}{B_0b(1-f_0)} << 1$.

Defining the half-lifetime t_B of the susceptible bacteria by $B(t_B) = B_0/2$, then

$$t_B = \frac{1}{B_0kb(1-f_0)}\log[\frac{B_0b(1-f_0)}{P_0}]. \quad [3]$$

While the calculations can be carried out with the full expressions (2) for $B(t)$ and $P(t)$, one can make the further simplifying approximation that $B(t)$ is practically constant ($= B_0$) for $t < t_B$ and zero afterwards. Within that approximation $P(t) = P_0e^{kbB_0(1-f_0)t}$. Note that $P(t_B) = B_0b(1-f_0)$, independent of the initial population of phage.

After t_B the phage die by infecting lysogens and are generated by induction:

$$\frac{dP}{dt} = -kL(t)P(t) + \gamma bL(t), \quad [4]$$

so the phage population reaches a steady state $P_{\text{ss}} = \gamma b/k$. Consequently the initial phage population in all subsequent dilution cycles will be $P_0 = \mu\gamma b/k$, where μ is the dilution ratio.

Lysogens and nutrient. With the above assumption that the susceptible bacteria are gone after time t_B, lysogens are only created by infections during $t < t_B$, and then exclusively grow on nutrient for $t > t_B$ at a rate α_L, until all the nutrient is consumed around $t = t_N$:

$$\frac{dL}{dt} = kf_0P(t)B(t) \simeq kf_0B_0P(t) = kf_0B_0P_0e^{kbB_0(1-f_0)t}, \quad t < t_B, \quad [5]$$

leading to

$$L(t < t_B) \simeq \frac{f_0P_0}{b(1-f_0)}(e^{kbB_0(1-f_0)t} - 1) = \frac{f_0}{b(1-f_0)}(P(t) - P_0). \quad [6]$$

Note that $L(t_B) \approx f_0B_0$, again independent of the initial concentration of phage. For $t > t_B$, $B(t > t_B) = 0$:

$$L(t > t_B) = L(t_B)e^{\alpha_L(t-t_B)} \simeq f_0B_0e^{\alpha_L(t-t_B)}. \quad [7]$$

For $t > t_B$, he lysogen concentration will grow until all nutrients are consumed at time t_N, defined by $N(t_N) \simeq K$:

$$\frac{dN}{dt} = -\frac{N}{N+K}(\alpha_B B + \alpha_L L). \quad [8]$$

We assume that $N(t) = N_0 \gg K$ for $t < t_N$ and $N(t) = 0$ afterwards, and that the nutrient is being consumed mainly by lysogens (for $t > t_B$), which leads to

$$\frac{dN}{dt} = -\alpha_L L(t) = -\alpha_L f_0 B_0 e^{\alpha_L(t-t_B)}, \quad t < t_N. \quad [9]$$

The solution is

$$N(t) = N_0 - f_0 B_0 e^{\alpha_L(t-t_B)} = N_0 - L(t). \quad [10]$$

Note that, by definition, $L(t_N) = N_0 - K \simeq N_0$. Solving for t_N, we find

$$t_N = \frac{1}{\alpha_L}\log\left(\frac{N_0}{f_0 B_0}\right) + t_B. \quad [11]$$

A comparison between the full numerical solution and these analytical results is presented in Fig. S1.

B. Invasion into a fixed strategy. We now check the stability of a given fixed strategy, coined the "major strategy", against invasion by another fixed strategy or an adapting strategy. To this end, we consider adding an infinitesimal density of the invading phage into the original (major) phage population, with $P_0^{\text{inv}} << P_0$. The times t_B and t_L are thus determined by the major strategy alone.

B.1. Invasion by a fixed strategy. For an invading phage with a fixed strategy $f = f_{\text{inv}}$, the concentrations of phage $P_a(t; f_{\text{inv}})$ and of lysogens $L_a(t; f_{\text{inv}})$ grow exponentially for $t < t_B$ at rates $kbB_0 f_{\text{inv}}$ and $kbB_0(1 - f_{\text{inv}})$, respectively. After the susceptible bacteria run out, the concentration of lysogen grows, as before, at a rate α_L:

$$\begin{aligned} L_a(t < t_B; f_{\text{inv}}) &= \frac{f_{\text{inv}} P_0^{\text{inv}}}{b(1-f_0)}(e^{kbB_0(1-f_{\text{inv}})t} - 1) \\ L_a(t_B < t < t_N; f_{\text{inv}}) &= \frac{f_{\text{inv}} P_0^{inv}}{b(1-f_{\text{inv}})}(e^{kbB_0(1-f_{\text{inv}})t_B} - 1)e^{\alpha_L(t-t_B)}. \end{aligned} \quad [12]$$

First, we find the optimal invading fixed strategy, by finding f_{inv} which maximizes Eq. 12:

$$\frac{dL_a(t_B; f_{\text{inv}})}{df_{\text{inv}}} = 0 \Rightarrow f^*_{\text{inv}}(f_0) = \frac{1}{2} - \sqrt{\frac{1}{4} - \frac{1-f_0}{\log(\frac{B_0 b(1-f_0)}{P_0})}}. \quad [13]$$

The overall optimal strategy f_{opt} is determined by $f^*_{\text{inv}}(f_{\text{opt}}) = f_{\text{opt}}$, which means that no other fixed strategy can invade it. This transcendental equation can be solved numerically, but if one assumes $\log(B_0 b/P_0) \gg \log(1 - f_{\text{opt}})$ then $f_{\text{opt}} \simeq 1/\log(B_0 b/P_0)$. For the biologically relevant parameters used in the simulations this gives $f_{\text{opt}} \simeq 0.08$, in good agreement with the simulations. In the following we assume that the fixed strategy is given by $f_0 = f_{\text{opt}}$.

B.2. Invasion by an adaptive strategy. An adaptive strategy (f_2, s_{th}) with initial phage population P_0^a will only make new phages and no lysogens until time t_s (determined by the signal and the value of s_{th}) and then switch to branching ratio f_2 between the lytic and lysogenic pathways. We allow the adaptive strategy to have a different lysogenic growth rate α_L^a and burst size b_a. Eqs. 12 become

$$\begin{aligned} & t_s < t < t_B : f = f_2 \\ & \qquad L_a(t) = \frac{f_2 P_a(t_s)}{(1-f_2)b_a}(e^{kB_0 b_a(1-f_2)(t-t_s)} - 1) \\ & t_B < t < t_N : \\ & \qquad L_a(t) = \frac{f_2 P_a(t_s)}{(1-f_2)b_a}(e^{kB_0 b_a(1-f_2)(t_B-t_s)} - 1)e^{\alpha_L^a(t-t_B)}. \end{aligned} \quad [14]$$

So at the end of the cycle, the number of invading lysogens is given by

$$L_a(t_N) = \frac{f_2}{(1-f_2)b_a} P_0^a e^{kB_0 b_a t_s}(e^{kB_0 b_a(1-f_2)(t_B-t_s)} - 1)e^{\alpha_L^a(t_N-t_B)}. \quad [15]$$

In order to find the optimal adaptive strategy we need to maximize $L_a(t_N)$ with respect to t_s and f_2. Finding the optimal t_s (as a function of f_2) and substituting it in Eq. 15, we find that $L_a(t_N)$ is a monotonically increasing function of f_2 between 0 and 1, so $f_2^{(\text{opt})} = 1$. The optimal t_s is then given by $t_s^{(\text{opt})} = t_B - 1/(kB_0 b_a)$ (one can also find s_{th} from this relation, but it is unnecessary for the rest of the calculations). Substituting $t_s = t_s^{(\text{opt})}$ into Eq. 15, we finally have

$$L_a(t_N)^{(\text{opt})} = \frac{P_0^a}{b_a} e^{kB_0 b_a t_B - 1} e^{\alpha_L^a(t_N-t_B)}. \quad [16]$$

C. Value of information. In order to find the advantage of the adaptive strategy, we compare the number of lysogens at the end of the cycle $L_a(t_N)$ for the optimal invading adaptive strategy to that number $L_0(t_N)$ for an invading fixed strategy with the same f_0 as the original optimal fixed strategy. Defining $G = L_a(t_N)/L_0(t_N)$, we have

$$G = \frac{(1-f_0)e^{kB_0 b_a t_B - 1} e^{\Delta\alpha_L (t_N - t_B)}}{f_0 \tilde{b}(e^{kbB_0(1-f_0)t_B} - 1)} \simeq \frac{P_0}{e f_0 b_a B_0} \left(\frac{B_0 b(1-f_0)}{P_0}\right)^{\tilde{b}/(1-f_0)} \left(\frac{N_0}{f_0 B_0}\right)^{\tilde{\alpha}_L^a - 1}, \qquad [17]$$

with $\tilde{b} = b_a/b$ and $\tilde{\alpha}_L^a = \alpha_L^a/\alpha_L$.

By definition, the critical values $\tilde{b}^*$ and $\tilde{\alpha}_L^*$ are the values which give $G = 1$. To find $\tilde{\alpha}_L^*$ we substitute $\tilde{b} = 1$:

$$\tilde{\alpha}_L^* = 1 + \frac{(1-f_0)\log(e f_0 b B_0/P_0) - \log(B_0 b(1-f_0)/P_0)}{(1-f_0)\log(N_0/f_0 B_0)}, \qquad [18]$$

and similarly to find $\tilde{b}$ we substitute $\tilde{\alpha}_L^* = 1$:

$$\tilde{b}^* = (1-f_0)\frac{\log(e f_0 b B_0 \tilde{b}^*/P_0)}{\log(B_0 b(1-f_0)/P_0)}. \qquad [19]$$

For the parameters used in the numerics, we find $\tilde{\alpha_L}^* \simeq 0.35$ and $\tilde{b}^* \simeq 0.8$, to be compared with the values $\tilde{\alpha_L}^* \simeq 0.44$ and $\tilde{b}^* \simeq 0.82$, obtained from the numerical simulations (Fig. 4 of the main text).

Note that the dependence on parameters in Eqs. (18) and (19) is logarithmic, which explains the robustness of the crossing-point values to parameters. For $\tilde{b}^*$ the ratio B_0/P_0 appears both in the numerator and the denominator, so the crossing point is practically insensitive to that parameter, while for $\tilde{\alpha_L}^*$ the ratio B_0/P_0 appears only at the numerator and has a slightly stronger influence on the crossing point.

D. Invasion into an adaptive strategy. In the above, we considered the invasion into the optimal fixed strategy by an adaptive strategy, and found the region of stability of that fixed strategy. We now consider the opposite scenario – invasion into the optimal adaptive strategy, with lysogenic growth rate α_L^a and burst size b_a, by a fixed strategy with possibly higher growth rate α_L or burst size b.

The only difference in the calculation is that for the adaptive strategy $(1, s_{\text{th}})$ new infections only generate phage for $t < t_s$ and only generate lysogens afterwards, where t_s is determined by the threshold s_{th}:

$$t_s = \frac{1}{k b_a B_0} \log\left(\frac{B_0 s_{\text{th}} b_a}{P_0(B_0 - s_{\text{th}})}\right). \qquad [20]$$

Thus, for $t < t_s$, $B(t)$ and $P(t)$ will be the same as for a resident fixed strategy with $f_0 = 0$. Assuming $t_s < t_B$ (where t_B is given by Eq. 3, with $f_0 = 0$), and the same type of approximations, we find for $t_s < t < t_B$

$$B(t) = B(t_s)e^{-kb_a P(t_s)(t-t_s)}$$
$$L(t) = \frac{b_a k B(t_s) s_{\text{th}} e^{\alpha_L^a (t-t_s)}}{(b_a k s_{\text{th}} + \alpha_L^a)}, \qquad [21]$$

where, by the solution for $t < t_s$ and the relation between t_s and s_{th}, we have $B(t_s) = B - s_{\text{th}}$ and $P(t_s) = b_a s_{\text{th}}$. For $t > t_B$ the number of lysogens will grow exponentially at a rate α_L^a up to the time t_N for the disappearance of nutrient, which is now given by

$$t_N = t_s + \frac{1}{\alpha_L^a} \log \frac{b_a k(B_0 + N_0 - s_{\text{th}})s_{\text{th}} + N_0 \alpha_L^a}{b_a k s_{\text{th}}(B_0 - s_{\text{th}})}. \qquad [22]$$

A comparison between the simulation and analytical result for the concentrations of phage and lysogens of an adapting strategy with $f_2 = 1$ and $s_{\text{th}} = 7$ is presented in Fig. S2, demonstrating that the analytical calculation captures the dynamics of the full numerical solution.

D.1. Invasion of an adaptive strategy by another adaptive strategy. In order to find the optimal adaptive strategy, we check for the invasion of an adaptive strategy with a smaller threshold signal s'_{th}, or, alternatively, smaller t'_s (a very similar calculation can be done for $t'_s > t_s$). The equation for the concentration of invading lysogens for $t > t'_s$ is given, as before, by

$$\frac{dL_{\text{inv}}(t; t'_s)}{dt} = kB(t)P_{\text{inv}}(t'_s) + \alpha_L^a L_{\text{inv}}(t; t'_s), \qquad [23]$$

where $P_{\text{inv}}(t = t'_s; t'_s)$ is the concentration of the invading phage at $t = t'_s$. Unlike before, the form of $B(t)$ is different for $t < t_s$ and for $t > t_s$ (for the latter it is given by Eq. 21). $L_{\text{inv}}(t; t'_s)$ can be obtained analytically and expressed in terms of hypergeometric functions (due to the cumbersomeness of the expression we do not quote it here). As before, the optimal strategy t_s (or the corresponding s_{th}) is such that $L_{\text{inv}}(t = t_N; t'_s)/L_{\text{inv}}(t = t_N; t_s) < 1$ for all $t'_s \neq t_s$. This optimal value can be calculated numerically. For the parameters quoted in the paper, we find $s_{\text{th}}^{\text{opt}} \sim 9.8$, close to the numerical value of $s_{\text{th}}^{\text{opt}} \sim 12$ obtained by direct competition between adaptive strategies.

D.2. Stability against invasion by a fixed strategy. We now check the stability of the optimal adaptive strategy against invasion by a fixed strategy, with possibly different lysogenic growth rate or burst size. As the calculation is very similar to the calculation above, we only quote the result:

$$L_{\mathrm{inv}}(t) \simeq \frac{P_0^{(\mathrm{inv})} f_{\mathrm{inv}}(e^{kbB_0(1-f_{\mathrm{inv}})t}-1)}{b(1-f_{\mathrm{inv}})}, \quad t < t_s. \qquad [24]$$

For $t > t_s$ $L_{\mathrm{inv}}(t)$ can again be calculated analytically and expressed in terms of hypergeometric functions. In the above f_{inv} is the lysogenic branching ratio of the invading fixed-strategy phage, and $P_0^{(\mathrm{inv})}$ their initial concentration. We now look at the ratio of L_{inv} at the end of the cycle to the concentration of invading phage with the optimal adaptive strategy, and check when this value becomes larger than unity, as a function of either b_a/b or α_L^a/α_L. For invasion into the optimal adaptive strategy, for the numerical values quoted in the paper, we find that the best fixed strategy can invade for either $\alpha_L^a/\alpha_L < 0.48$ or $b_a/b < 0.8$, again close to the values found in the full numerical simulations.

2. Additional calculations

A. The optimal value of f_1. Strategies were initially defined by three parameters: $(f_1, f_2, s_{\mathrm{th}})$. Searching for the optimal strategy (i.e., the strategy that maximizes the number of lysogen cells at the end of a series of dilution cycles) on a dense grid of parameters, we find that for all the optimal strategies $f_1 = 0$, as shown in Fig. S4. Hence, the possible optimal strategies are now defined by only two free parameters (f_2, s_{th}).

B. Graded response to signal. In the model we consider that transition between $f = 0$ and $f = f_2$ for an adaptive strategy to be abrupt and to occur when $s = s_{\mathrm{st}}$. We also tested a graded response, of the form:

$$f(s) = \frac{1}{e^{-\frac{s-s_{\mathrm{th}}}{w}}+1}, \qquad [25]$$

i.e. the transition of the lysogen branching ratio occurs over a scale of w in signal level. As shown in Fig. S5 implementing a graded transition does not produce a significant change in the results. Specifically, the value of information remains unchanged, even when w changes by orders of magnitude. Only when w becomes comparable to the threshold s_{th} itself, namely when the adaptive strategy does not appreciably change over the range of ambient signal concentrations, then the crossing-point lysogen growth rate reverts to the original growth rate, i.e. the value of information becomes negligible.

C. Signal release by a dormant phage. Ref. (1) showed that a dormant phage (i.e., a phage that has been incorporated into a lysogen cell) can also release the signal peptide arbitrium. To take this effect into account, we added a corresponding term to Eq. 5:

$$\underbrace{\frac{ds}{dt}}_{\text{Signal}} = \underbrace{kB\sum_i P_i(1-f_i)}_{\text{Secreted upon lytic infection}} + \underbrace{(\gamma+\beta)\sum_i L_i}_{\text{Secreted upon induction and constitutively}}, \qquad [26]$$

where β stands for the rate at which lysogens secrete signal (note that since γ, the lysogen induction rate, also appears elsewhere in the equations, this is not a mere change in the value of γ). Fig. S6 depicts the dependence of the crossing-point values of α_L^* and b^* on the rate β. For the wide range of relevant rates, this term has little effect on the crossing-point values.

D. Phage decay rate. In our model, we assume that phage die when trying to infect a lysogenic cell. This is well established (2). Nevertheless, we checked what happens when we replace the death of phage upon infecting a lysogen with the standard decay rate of phage. We find that this does not change the result, not even quantitatively. Fig. S7 A and B depict what happens when we discard completely the term corresponding to phage death upon attempting to infect a lysogen cell, but instead take a fixed phage decay rate of 0.8 hour^{-1}. Very similar results were found when taking the decay rate of phage to be 10^{-3} (hour)$^{-1}$ (3) together with a 100% chance of phage death upon infecting a lysogen, or taking a decay rate of 0.1 (hour)$^{-1}$ (4) together with a 50% chance of phage death upon infecting a lysogen. The system's dynamics and the crossing-point values for both the burst size and growth rate were unaffected. This is easily understandable, as the number of lysogens is almost constant through most of the cycle, so the two scenarios for phage death/decay are almost equivalent.

E. Lytic infection with time delay. In our model, we assume an instantaneous lytic cycle, i.e., we take the lysis time between the start of a lytic infection and actual cell lysis to be much shorter than all other time scales (the same assumption is made in e.g. Refs.(5–7)). To check if our results change when eliminating this assumption, we extended our model to include a lysis delay time τ. In this case, we introduce new species B_i, bacteria that were infected by strategy i, and, after the delay, will burst and produce phage P_i. In the same spirit, the induced lysogens will also turn into B_i before the burst. The equations now become:

$$\frac{dB}{dt} = \alpha_B B \frac{N}{N+K} - kB \sum_i P_i \quad [27]$$

$$\frac{dL_i}{dt} = \alpha_L L_i \frac{N}{N+K} + f_i kBP_i - \gamma L_i \quad [28]$$

$$\frac{dB_i}{dt} = k\Bigg([1 - f_i(t)]B(t)P_i(t) - [1 - f_i(t-\tau)]B(t-\tau)P_i(t-\tau) \Bigg) + \gamma \Bigg(L_i(t) - L_i(t-\tau) \Bigg) \quad [29]$$

$$\frac{dP_i}{dt} = [1 - f_i(t-\tau)]bkB(t-\tau)P_i(t-\tau) - kP_i \left(B + \sum_i (L_i + B_i) \right) + \gamma b L_i(t-\tau) \quad [30]$$

$$\frac{dN}{dt} = -\frac{N}{N+K} \left(\alpha_B B + \alpha_L \sum_i L_i \right) \quad [31]$$

$$\frac{ds}{dt} = kB(t-\tau) \sum_i [1 - f_i(t-\tau)]P_i(t-\tau), \quad [32]$$

where all quantities without an explicit time variable are evaluated at time t.

Fig. S8 shows the critical burst size and critical growth rate, both normalized to the fixed strategies parameters, for different time delays. As reported in (8), a burst size of $b \approx 10$ is a reasonable choice for a delay time of 0.5 (hour). The delay slows down the creation of phages, hence allowing the bacteria to grow for a longer time, and devour all the nutrients. In order to prevent a situation of the nutrient running out before the lysogens appear, we increased the initial nutrient concentration in the first cycle to $N_0 = 10^{12}$ bacterial equivalents mL^{-1}. As shown in Fig. S8 the crossing-points values remain approximately the same as in the case without lytic delay.

References

1. N Aframian, et al., Dormant phages communicate via arbitrium to control exit from lysogeny. *Nat. Microbiol.* **7**, 145–153 (2022).
2. S Brown, N Mitarai, K Sneppen, Protection of bacteriophage-sensitive escherichia coli by lysogens. *Proc. Natl. Acad. Sci. United States Am.* **119**, e2106005119 (2022).
3. H Doekes, G Mulder, R Hermsen, Repeated outbreaks drive the evolution of bacteriophage communication. *eLife* **10**, e58410 (2021).
4. RS Eriksen, N Mitarai, K Sneppen, Sustainability of spatially distributed bacteria-phage systems. *Sci. Reports* **10**, 3154 (2020).
5. V Sinha, A Goyal, S Svenningsen, S Semsey, S Krishna, In silico Evolution of Lysis-Lysogeny Strategies Reproduces Observed Lysogeny Propensities in Temperate Bacteriophages. *Front Microbiol* **8**, 1386 (2017).
6. L Wahl, M Betti, D Dick, T Pattenden, A Puccini, volutionary stability of the lysis-lysogeny decision: Why be virulent? *Evol* **73**, 92–98 (2019).
7. BA Berryhill, et al., The book of lambda does not tell us that naturally occurring lysogens of <i>escherichia coli</i> are likely to be resistant as well as immune. *Proc. Natl. Acad. Sci.* **120**, e2212121120 (2023).
8. I Wang, Lysis timing and bacteriophage fitness. *Genetics* **172**, 17–26 (2006).

3. Figures caption

- Fig. S1: Comparison between the simulation (solid curve) and analytical (dashed curve) results, for a fixed strategy $f_0 = 0.1$. The blue and orange curves are the phage and lysogens, respectively, of the resident strategy, both in units of bacterial equivalents mL^{-1}.

- Fig. S2: Comparison between the simulation (solid curve) and analytical (dashed curve) results, for the adaptive strategy $(f_2, s_{\text{th}}) = (1, 7)$. The blue and orange curves are the phage and lysogens, respectively, both in units of bacterial equivalents mL^{-1}.

- Fig. S3: Lysogens fraction at $b_a = b^* = 12.4$ (blue) and at $\alpha_L a = \alpha_L^* = 0.31$ for different initial nutrient (N_0). Showing a wide range of coexistence of adaptive and fixed strategies near the growth rate crossing point, and a narrow range near the burst size crossing point (Note that the x-axis is in a logarithmic scale).

- Fig. S4: $f_1 = 0$ is optimal for lysogen maximization. A set of strategies $(f_1, f_2, s_{\mathbf{th}})$ where $f_1, f_2 \in [0, 1]$ and $s_{\mathbf{th}} \in [0, 20]$ were were simultaneously competed in a simulation. The histogram shows the number of lysogens for the 10 best strategies (the largest number of lysogens at the last dilution cycle). All 10 have $f_1 = 0$.

- Fig. S5: Crossing-point burst size (A) and growth rate (B) as functions of signal-response range w, where $f(s) = \frac{1}{e^{-\frac{s-s_{\rm th}}{w}}+1}$. For $w << s_{\rm th}$ no significant change is observed ($\alpha_L^*/\alpha_{L0} \approx 0.5$ and $b_a^*/b_0 \approx 0.8$).
- Fig. S6: The value of information in burst size (A) and growth rate (B), as a function of the signals release rate by lysogen cells, β. There is no change in the results
- Fig. S7: The crossing-point values of both burst (A) size and growth rate (B) remain approximately unchanged when replacing the death of phage upon infecting a lysogen with a constant phage death/decay rate of $0.8\,\mathrm{hour}^{-1}$.
- Fig. S8: Crossing-point values of burst size (A) and growth rate (B). No significant change in the result, when compensating the slow phage creation, and hence high bacterial growth, with higher concentration of nutrient.

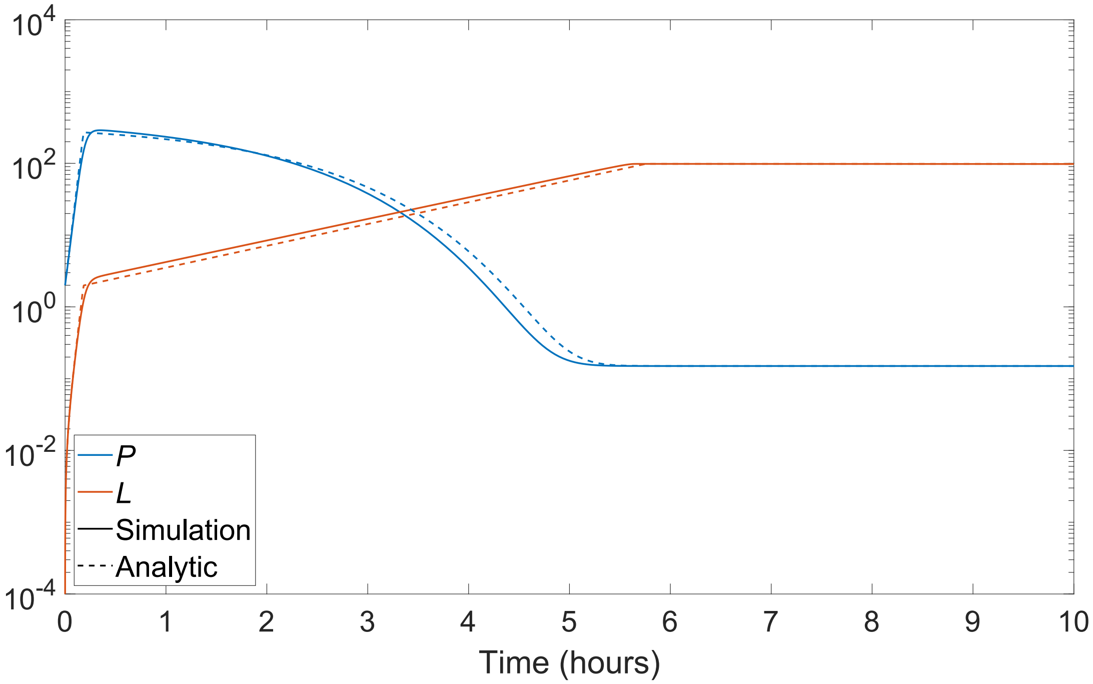

Fig. S1. Comparison between the simulation (solid curve) and analytical (dashed curve) results, for a fixed strategy $f_0 = 0.1$. The blue and orange curves are the phage and lysogens, respectively, of the resident strategy, both in units of bacterial equivalents mL^{-1}.

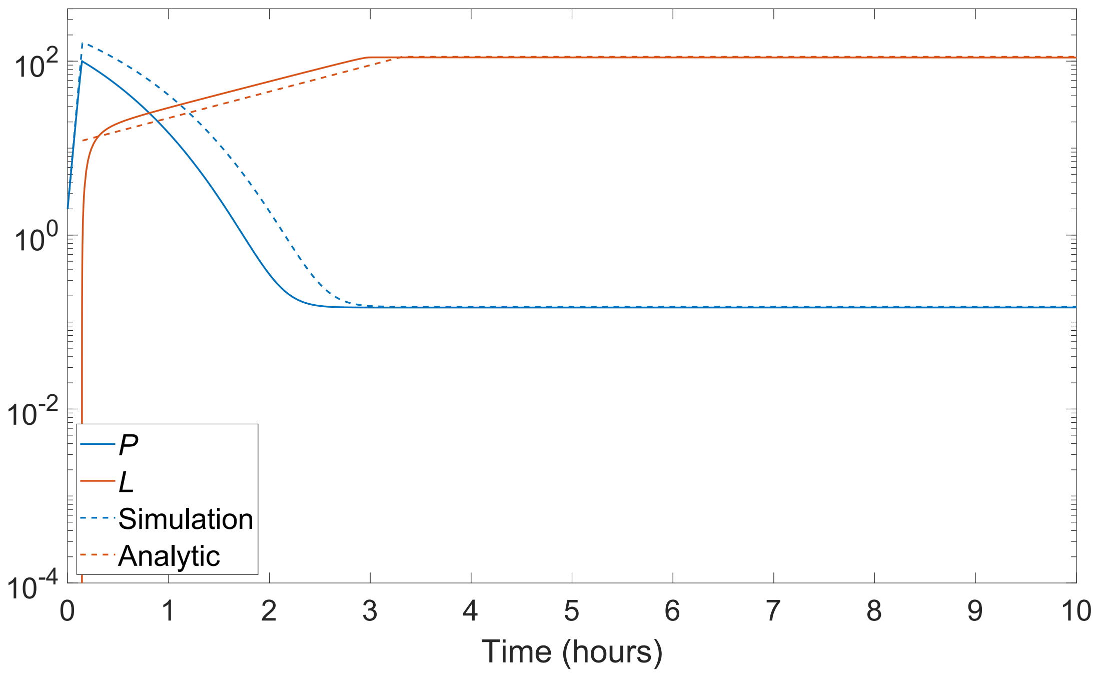

Fig. S2. Comparison between the simulation (solid curve) and analytical (dashed curve) results, for the adaptive strategy $(f_2, s_{\rm th}) = (1, 7)$. The blue and orange curves are the phage and lysogens, respectively, both in units of bacterial equivalents mL^{-1}.

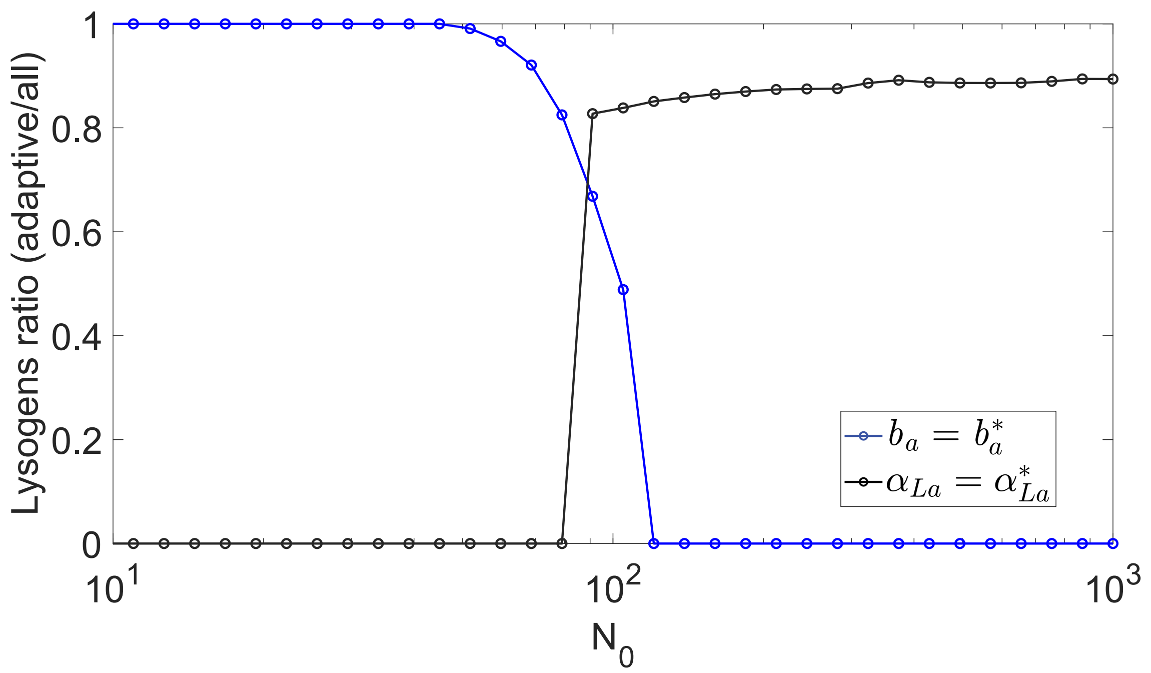

Fig. S3. Lysogens fraction at $b_a = b^* = 12.4$ (blue) and at $\alpha_L a = \alpha_L^* = 0.31$ for different initial nutrient (N_0). Showing a wide range of coexistence of adaptive and fixed strategies near the growth rate crossing point, and a narrow range near the burst size crossing point (Note that the x-axis is in a logarithmic scale).

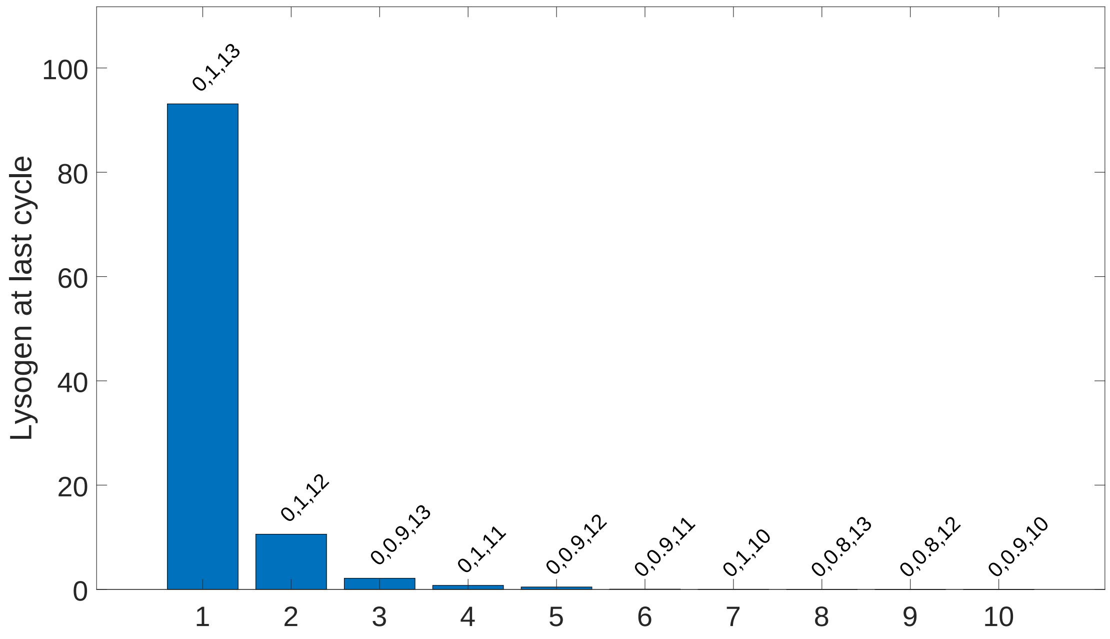

Fig. S4. $f_1 = 0$ is optimal for lysogen maximization. A set of strategies $(f_1, f_2, s_{\textbf{th}})$ where $f_1, f_2 \in [0, 1]$ and $s_{\textbf{th}} \in [0, 20]$ were were simultaneously competed in a simulation. The histogram shows the number of lysogens for the 10 best strategies (the largest number of lysogens at the last dilution cycle). All 10 have $f_1 = 0$.

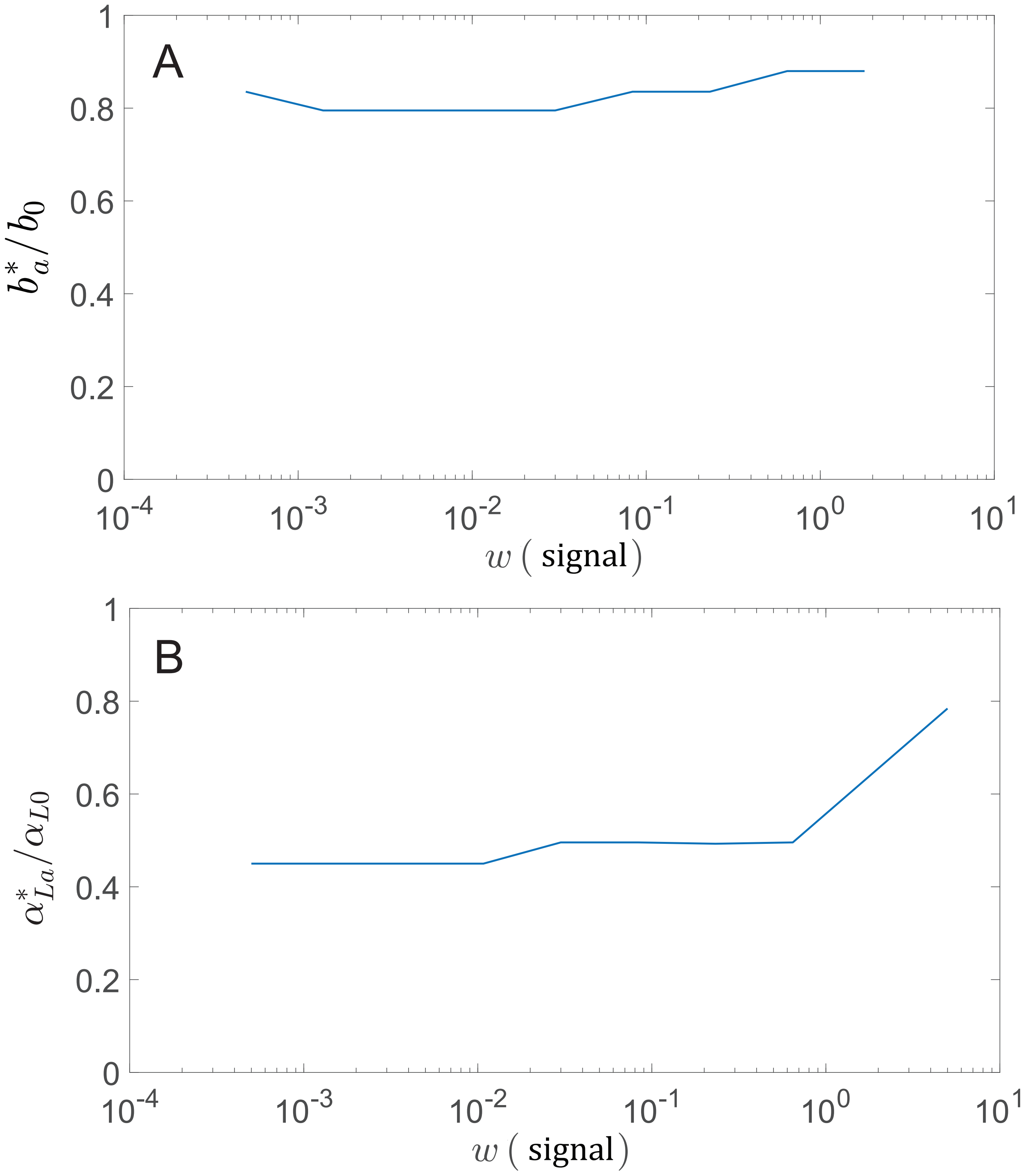

Fig. S5. Crossing-point burst size (A) and growth rate (B) as functions of signal-response range w, where $f(s) = \frac{1}{e^{-\frac{s-s_{\text{th}}}{w}}+1}$. For $w << s_{\text{th}}$ no significant change is observed ($\alpha_L^*/\alpha_{L0} \approx 0.5$ and $b_a^*/b_0 \approx 0.8$)

.

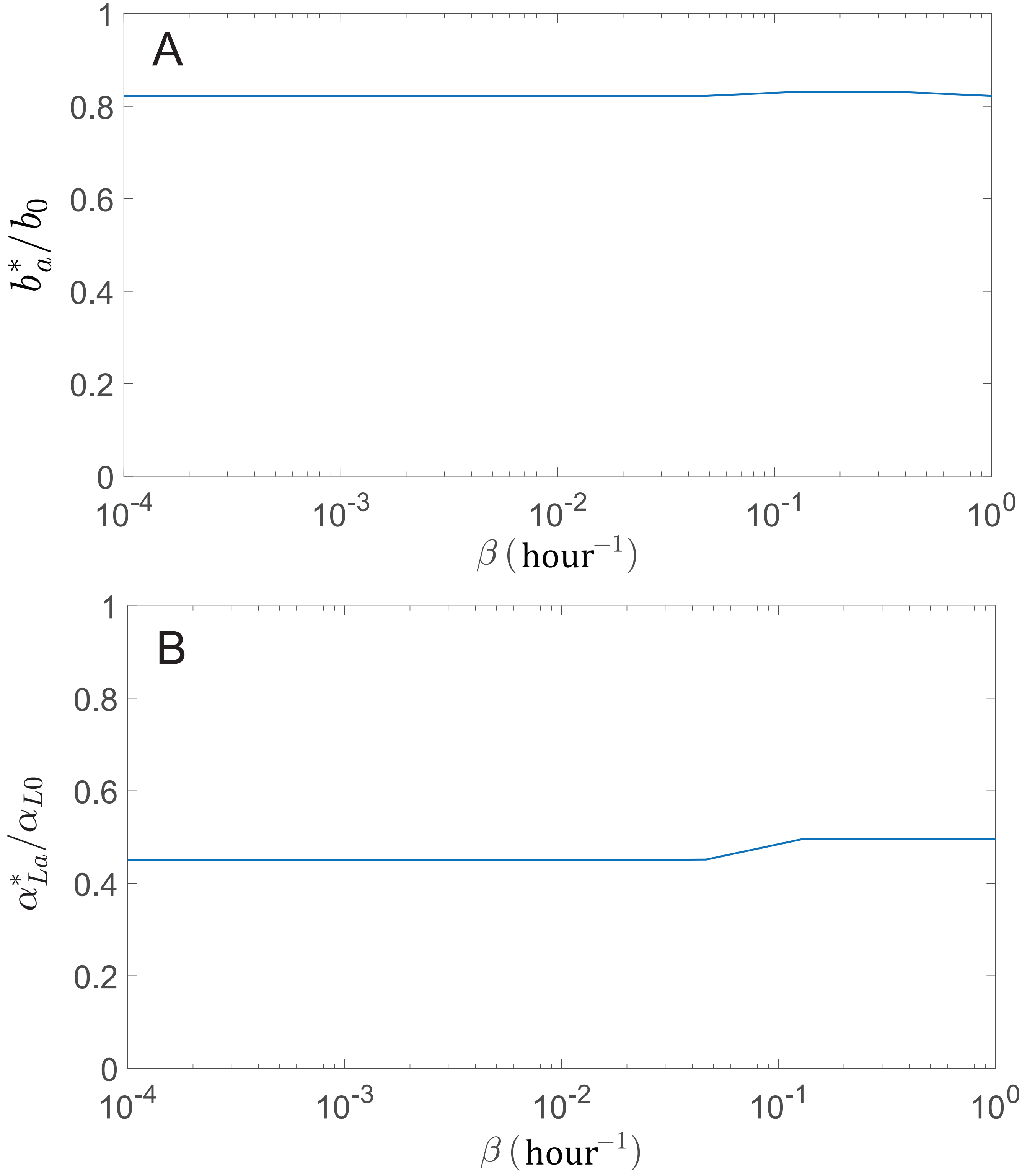

Fig. S6. The value of information in burst size (A) and growth rate (B), as a function of the signals release rate by lysogen cells, β. There is no change in the results

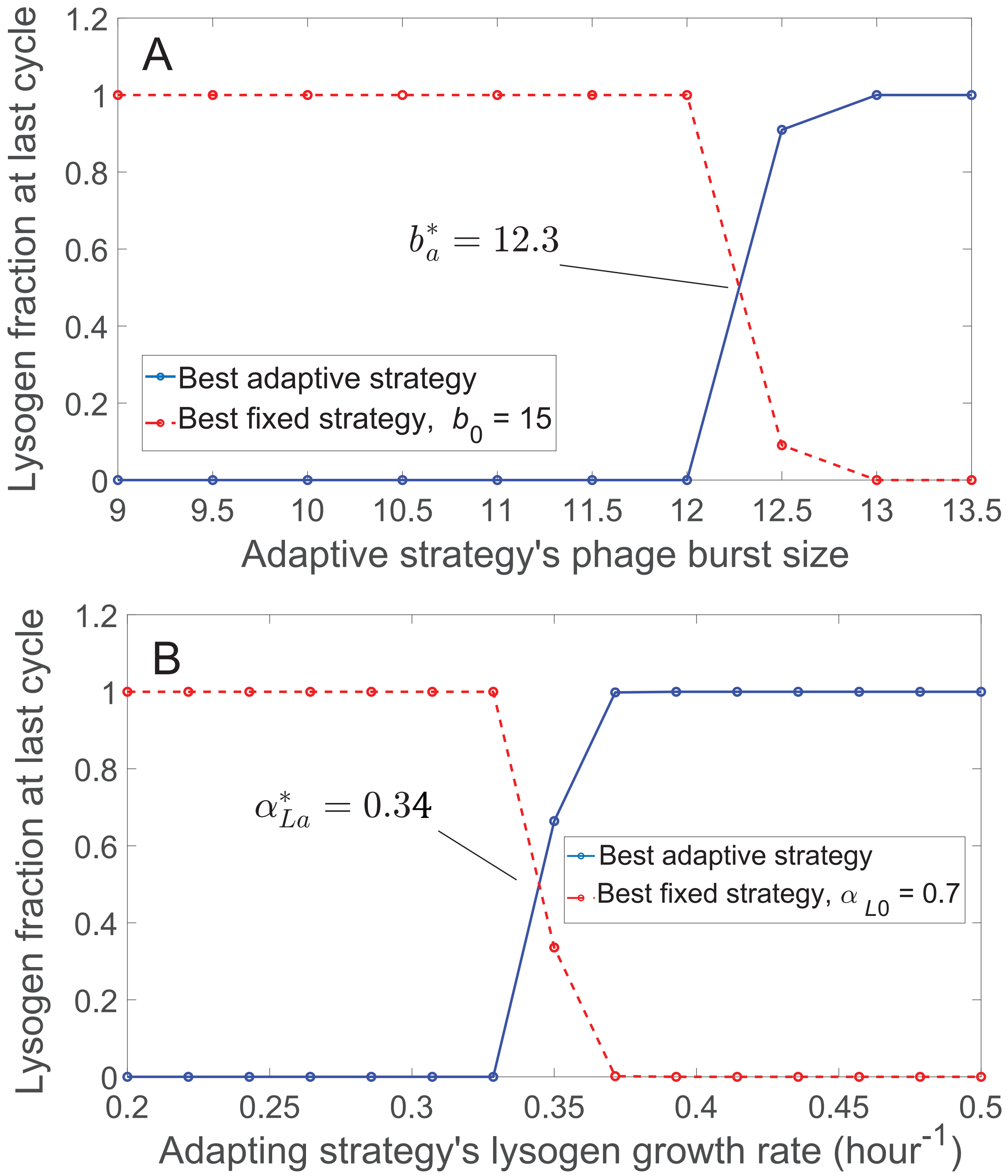

Fig. S7. The crossing-point values of both burst (A) size and growth rate (B) remain approximately unchanged when replacing the death of phage upon infecting a lysogen with a constant phage death/decay rate of $0.8\,\mathrm{hour}^{-1}$.

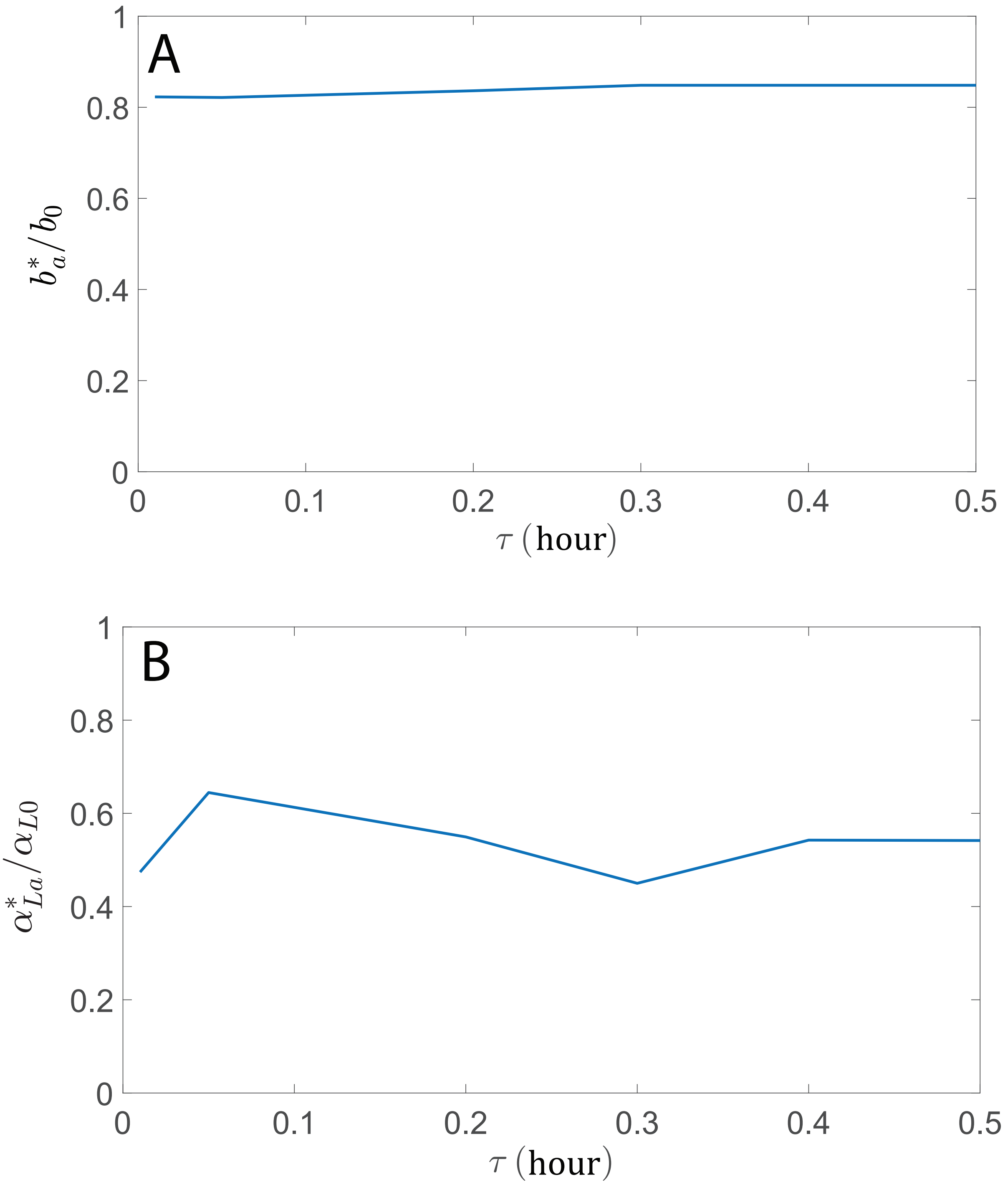

Fig. S8. Crossing-point values of burst size (A) and growth rate (B). No significant change in the result, when compensating the slow phage creation, and hence high bacterial growth, with higher concentration of nutrient
