# Supplementary figures and images for: The value of information gathering in phage–bacteria warfare
Source: PNAS Nexus. 2024 Jan 9;3(1):pgad431. doi: 10.1093/pnasnexus/pgad431 (PMC10776245; doi:10.1093/pnasnexus/pgad431)

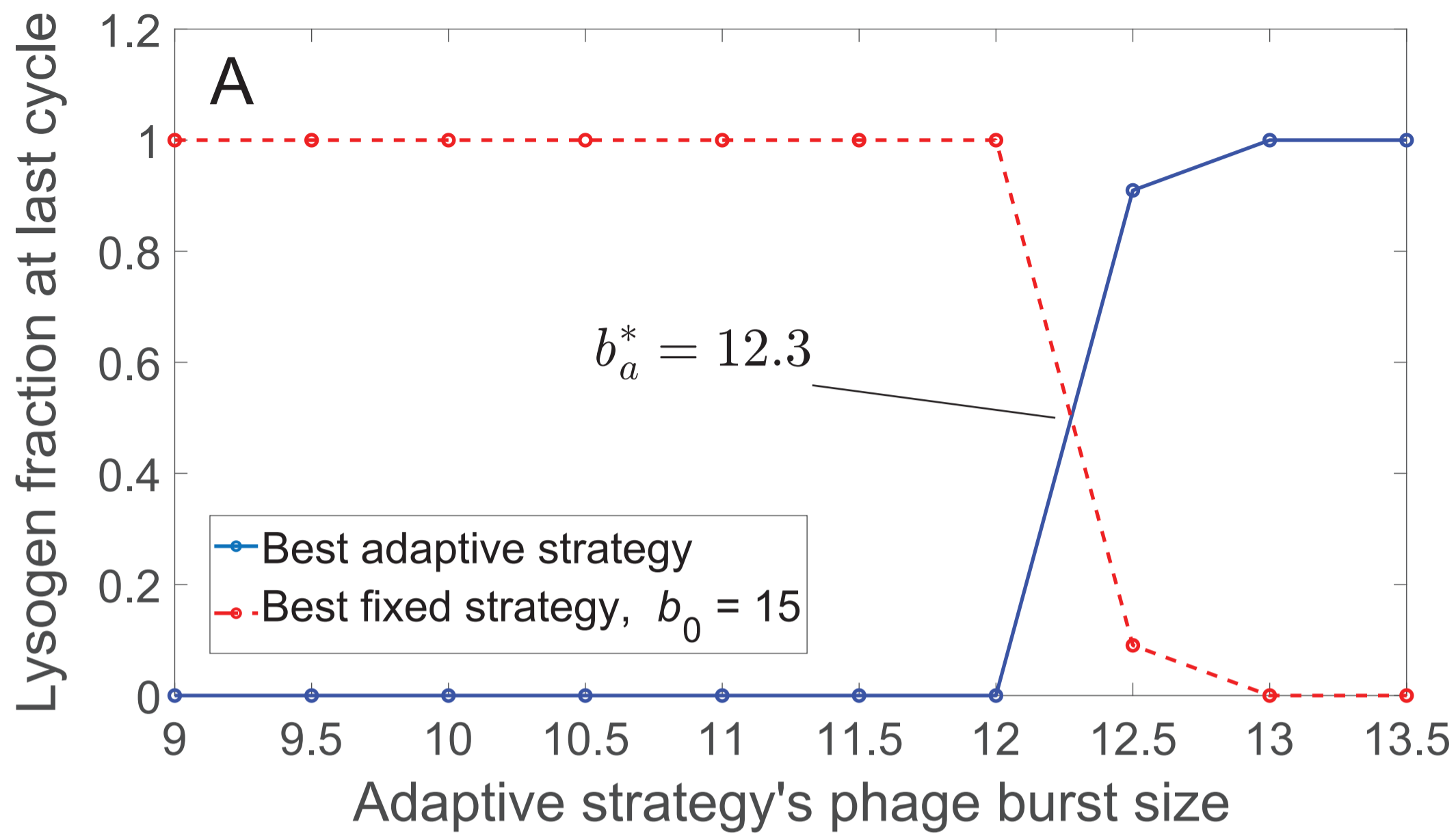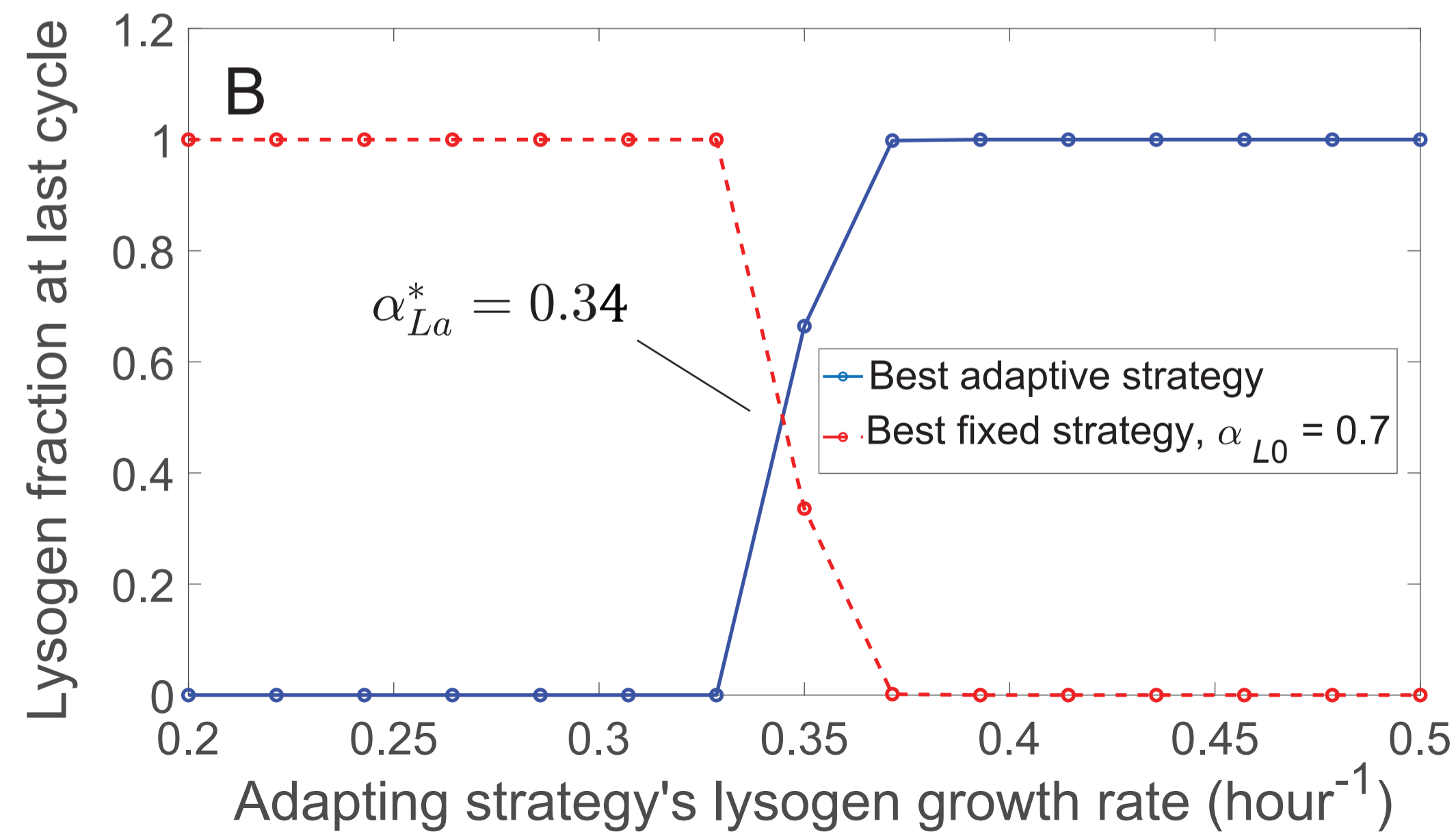

Supplement: pgad431_Supplementary_Data [file pgad431_supplementary_data.zip › PNASNEXUS-PNASNEXUS-2023-00941R-s04.pdf]

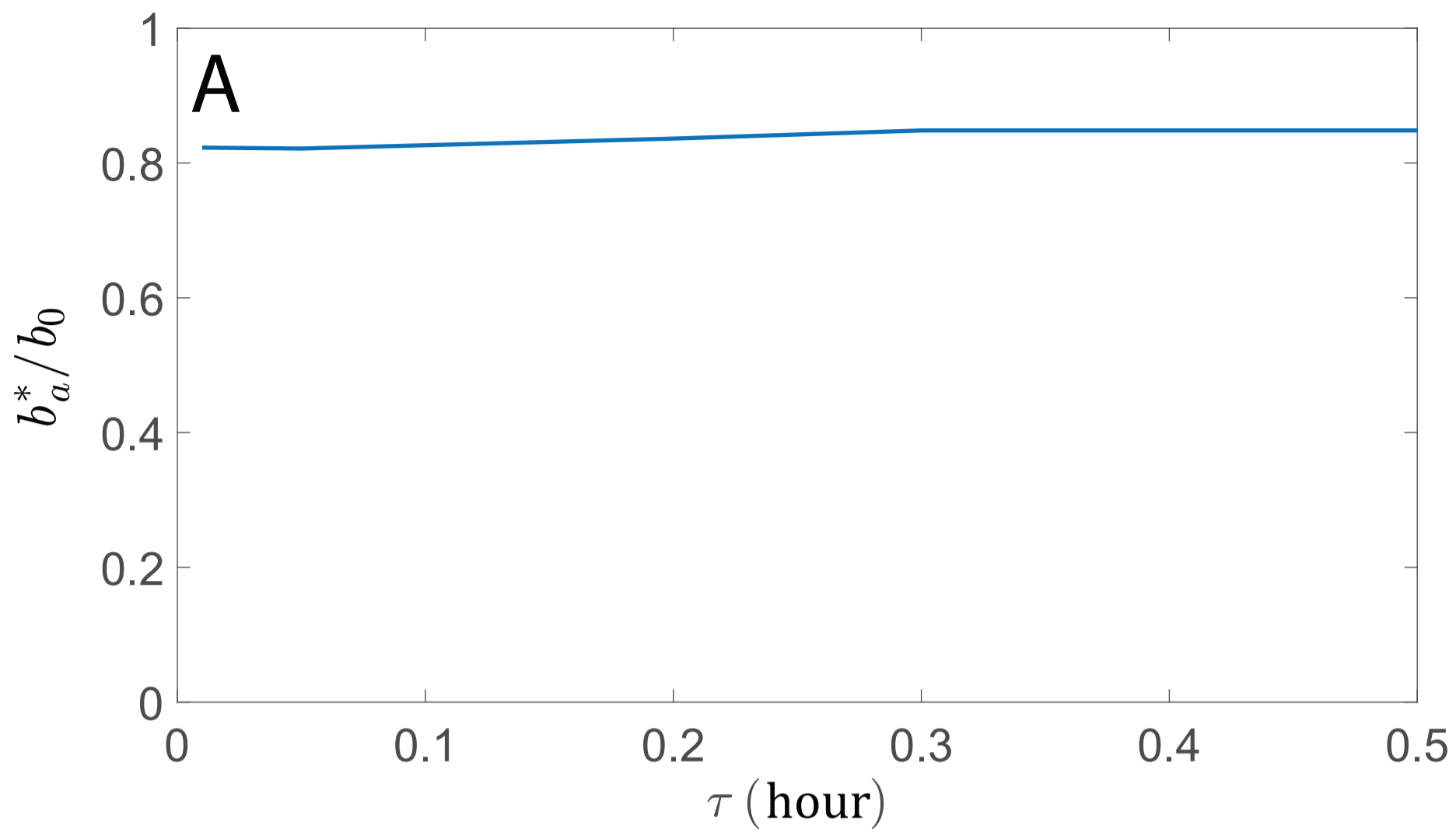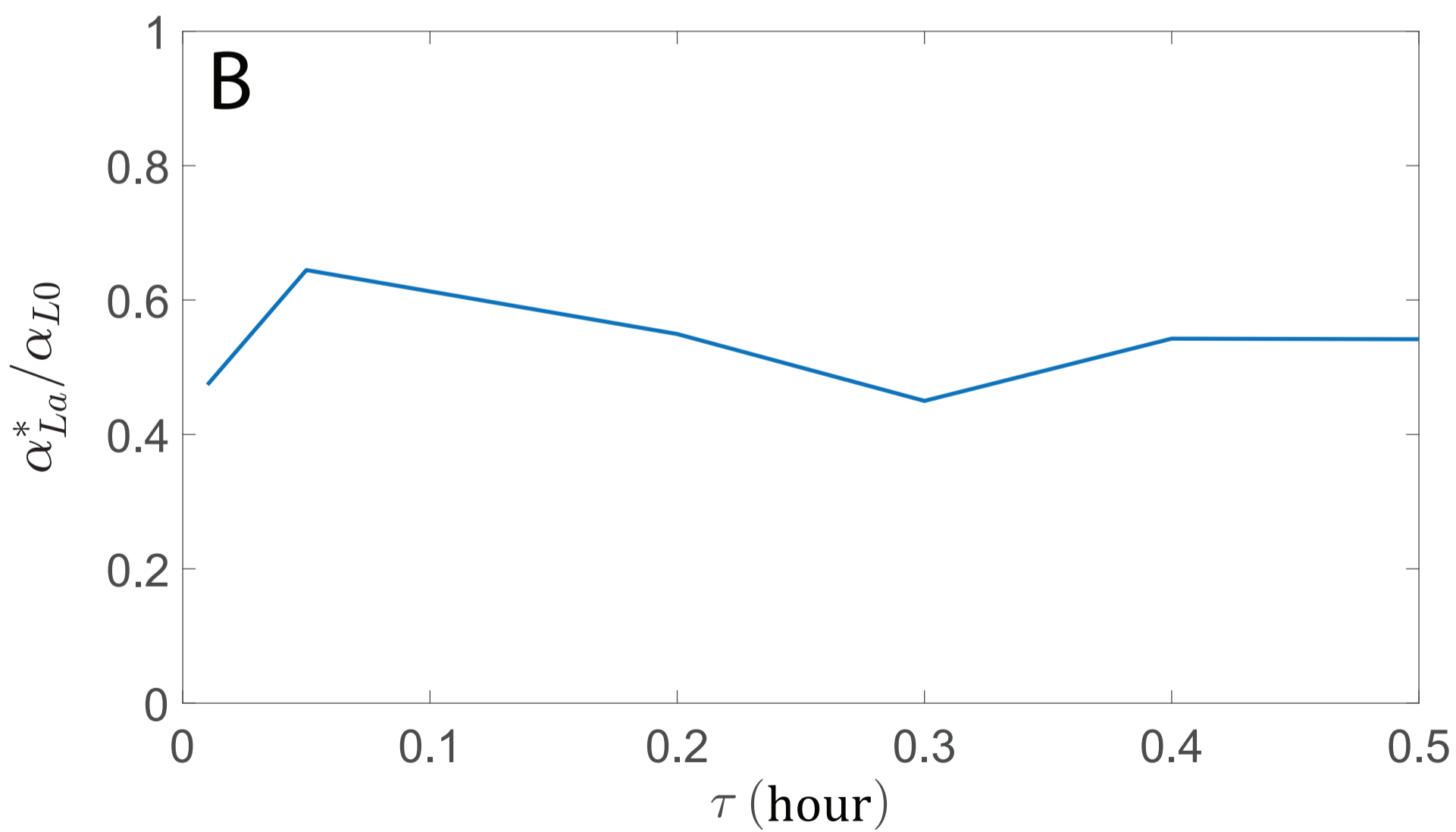

Supplement: pgad431_Supplementary_Data [file pgad431_supplementary_data.zip › PNASNEXUS-PNASNEXUS-2023-00941R-s05.pdf]

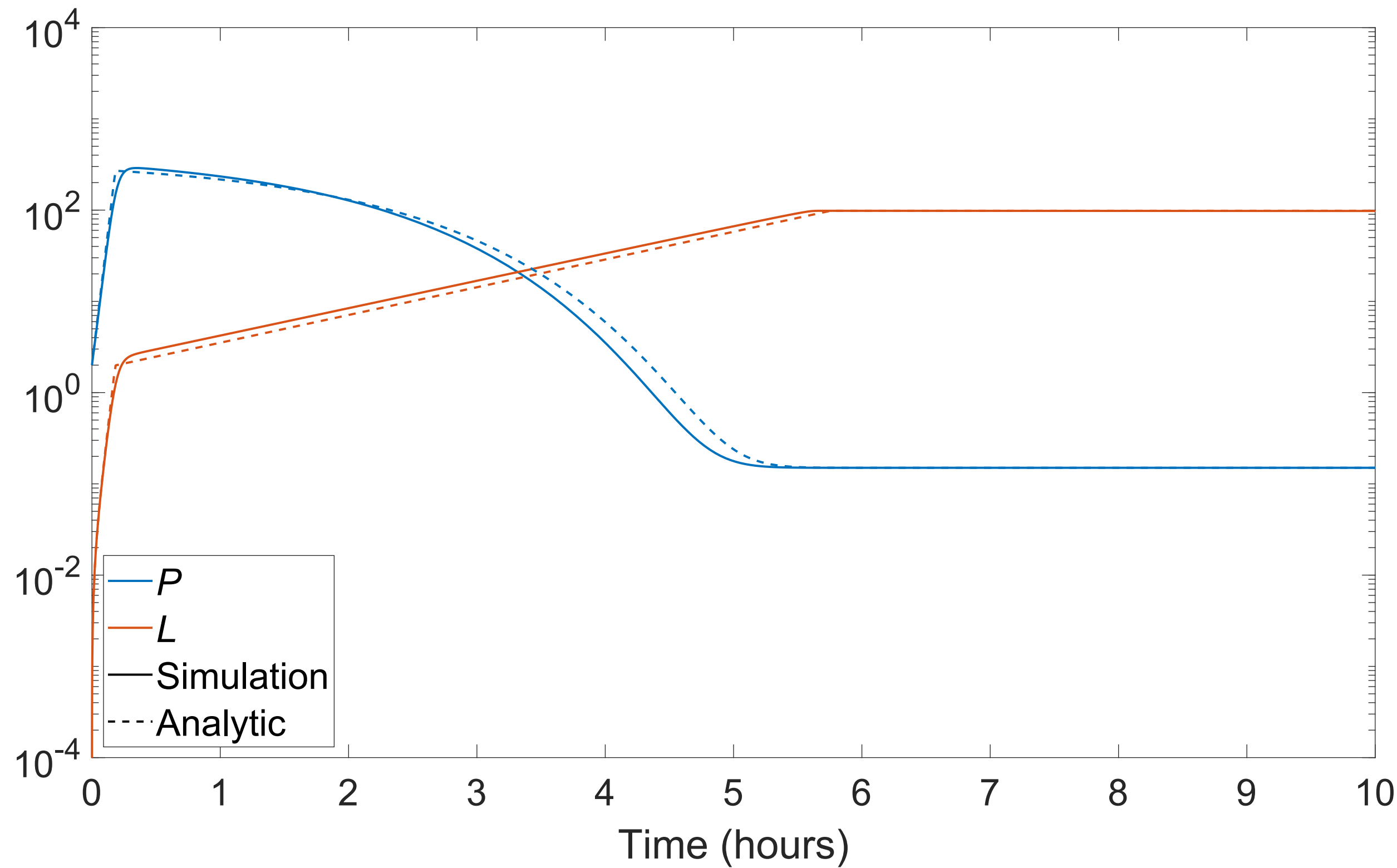

Supplement: pgad431_Supplementary_Data [file pgad431_supplementary_data.zip › PNASNEXUS-PNASNEXUS-2023-00941R-s06.pdf]

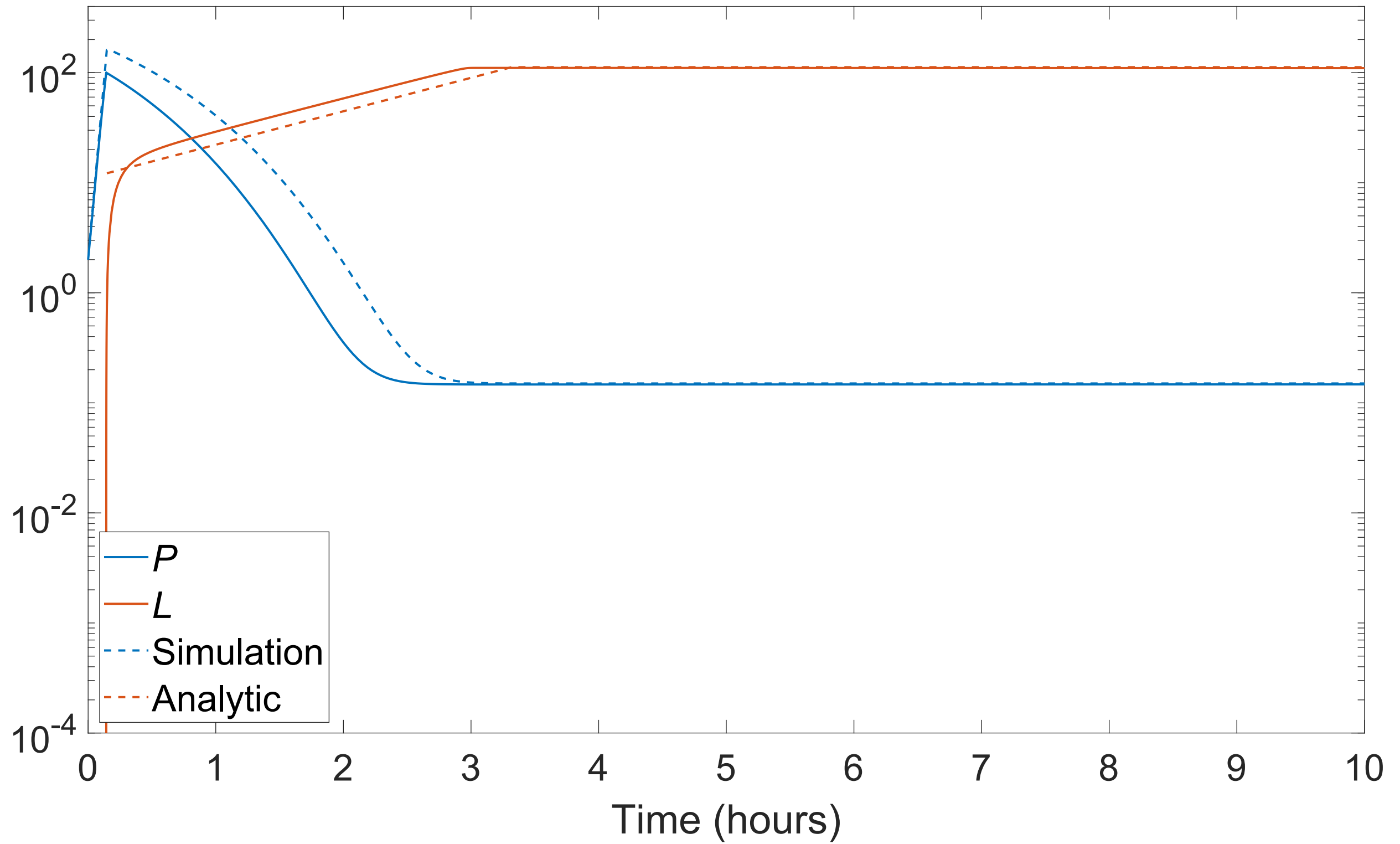

Supplement: pgad431_Supplementary_Data [file pgad431_supplementary_data.zip › PNASNEXUS-PNASNEXUS-2023-00941R-s07.pdf]

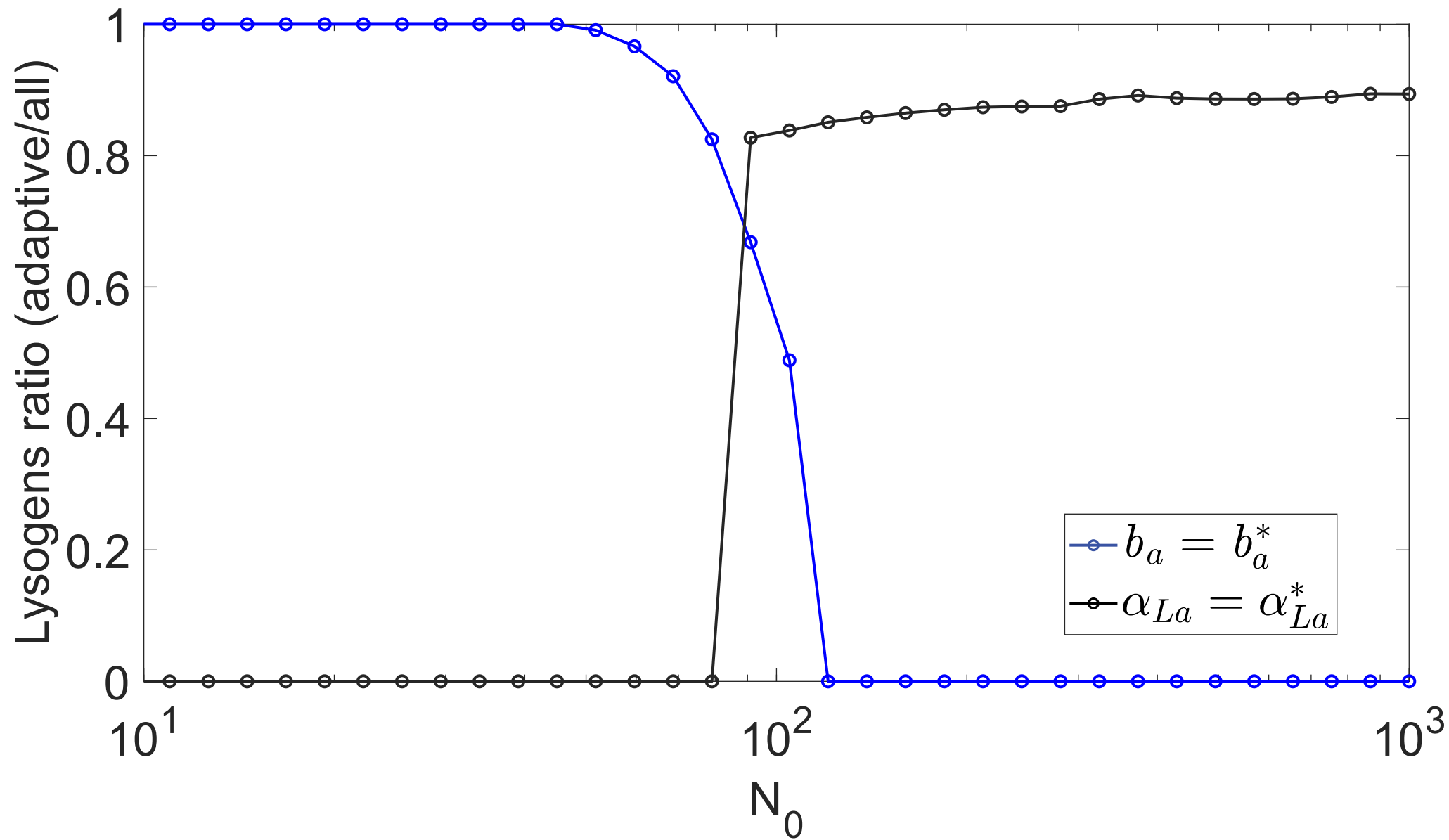

Supplement: pgad431_Supplementary_Data [file pgad431_supplementary_data.zip › PNASNEXUS-PNASNEXUS-2023-00941R-s08.pdf]

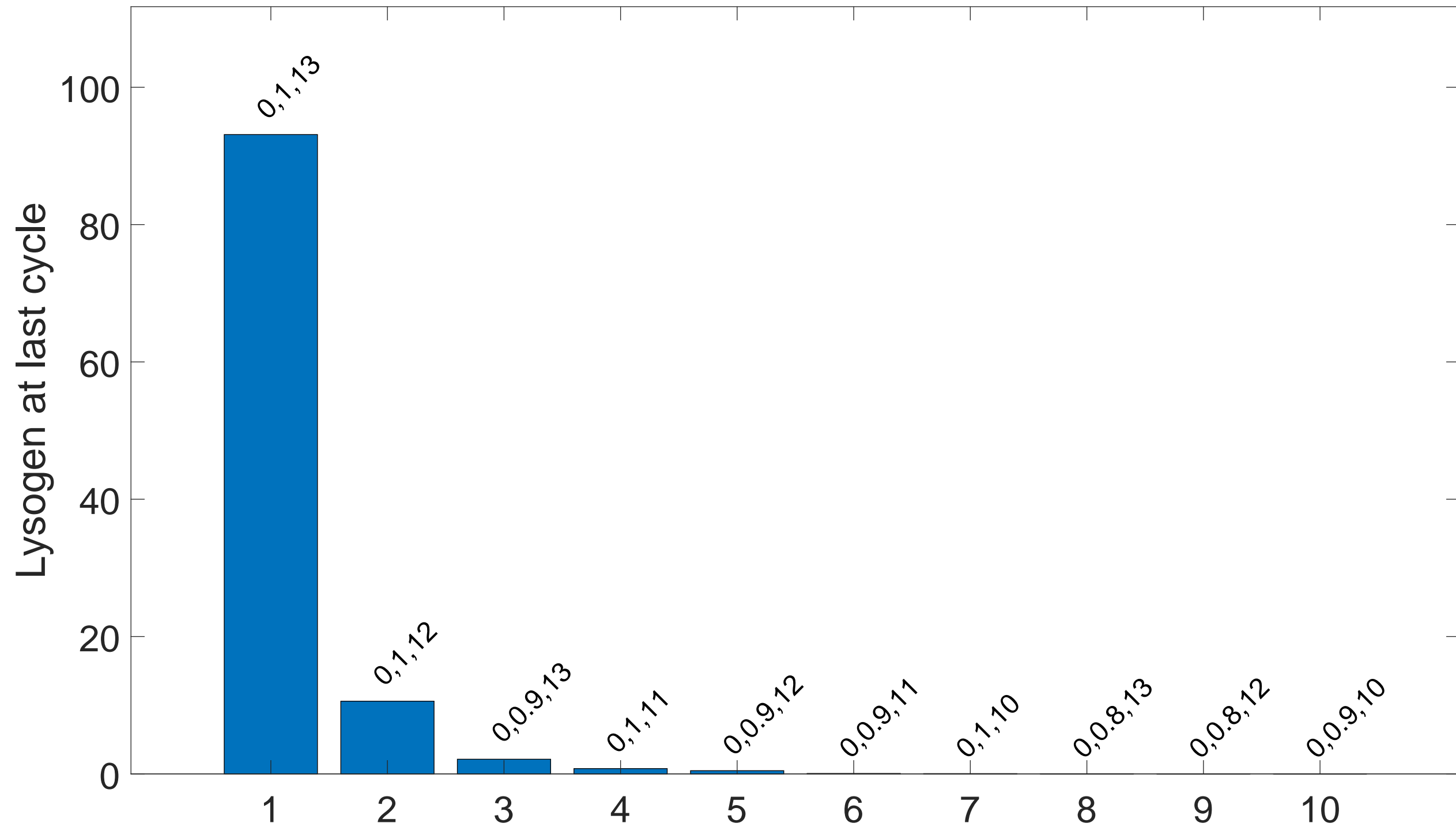

Supplement: pgad431_Supplementary_Data [file pgad431_supplementary_data.zip › PNASNEXUS-PNASNEXUS-2023-00941R-s09.pdf]

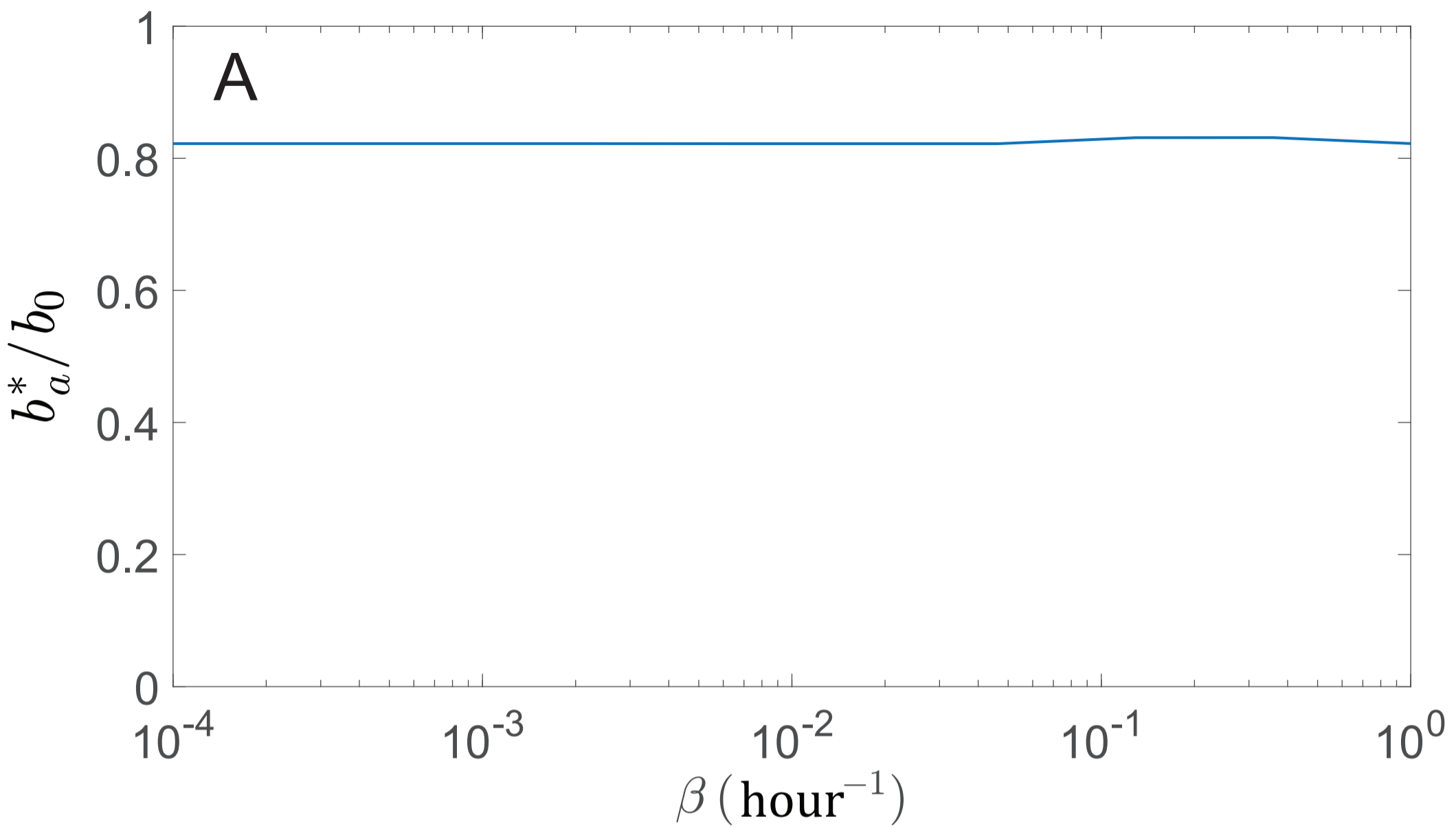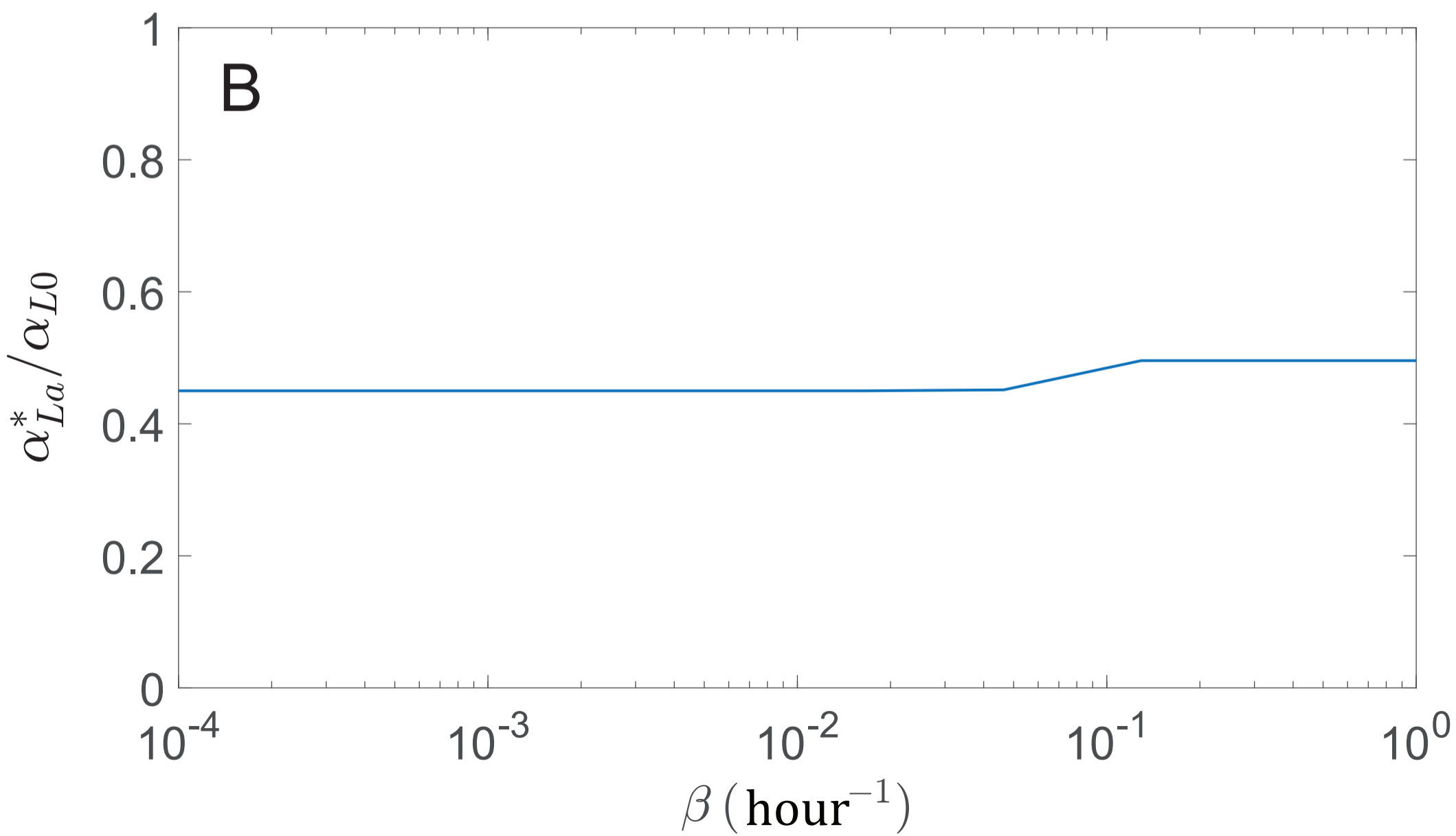

Supplement: pgad431_Supplementary_Data [file pgad431_supplementary_data.zip › PNASNEXUS-PNASNEXUS-2023-00941R-s03.pdf]
